# Repetitive transcranial magnetic stimulation alleviates motor impairment in Parkinson’s disease: association with peripheral inflammatory regulatory T-cells and SYT6

**DOI:** 10.1186/s13024-024-00770-4

**Published:** 2024-10-25

**Authors:** Fen Xie, Bibiao Shen, Yuqi Luo, Hang Zhou, Zhenchao Xie, Shuzhen Zhu, Xiaobo Wei, Zihan Chang, Zhaohua Zhu, Changhai Ding, Kunlin Jin, Chengwu Yang, Lucia Batzu, K Ray Chaudhuri, Ling-Ling Chan, Eng-King Tan, Qing Wang

**Affiliations:** 1 Department of Neurology, Zhujiang Hospital of Southern Medical University, Guangzhou, Guangdong 510282 People’s Republic of China; 2 Clinical Research Centre, Orthopedic Centre, Zhujiang Hospital of Southern Medical University, Guangzhou, Guangdong 510282 People’s Republic of China; 3 Department of Pharmacology and Neuroscience, University of North Texas Health Science Center, Fort Worth, TX 76107 USA; 4 Division of Biostatistics and Health Services Research, Department of Population and Quantitative Health Sciences, T. H. Chan School of Medicine, UMass Chan Medical School, Worcester, MA 01605 USA; 5 Parkinson Foundation International Centre of Excellence at King’s College Hospital, and Kings College, Denmark Hill, London, SE5 9RS UK; 6 Department of Neurology, National Neuroscience Institute, Singapore, Singapore; 7 7Singapore General Hospital, Singapore; Duke-NUS Medical School, Singapore, Singapore

**Keywords:** Parkinson’s disease, Repetitive transcranial magnetic stimulation, Regulatory T cells, Neuro-inflammation, Motor dysfunction, syt6

## Abstract

**Background:**

Repetitive transcranial magnetic stimulation (rTMS) has been used to treat various neurological disorders. However, the molecular mechanism underlying the therapeutic effect of rTMS on Parkinson’s disease (PD) has not been fully elucidated. Neuroinflammation like regulatory T-cells (Tregs) appears to be a key modulator of disease progression in PD. If rTMS affects the peripheral Tregs in PD remains unknown.

**Methods:**

Here, we conducted a prospective clinical study (Chinese ClinicalTrials. gov: ChiCTR 2100051140) involving 54 PD patients who received 10-day rTMS (10 Hz) stimulation on the primary motor cortex (M1) region or sham treatment. Clinical and function assessment as well as flow cytology study were undertaken in 54 PD patients who were consecutively recruited from the department of neurology at Zhujiang hospital between September 2021 and January 2022. Subsequently, we implemented flow cytometry analysis to examine the Tregs population in spleen of MPTP-induced PD mice that received rTMS or sham treatment, along with quantitative proteomic approach reveal novel molecular targets for Parkinson's disease, and finally, the RNA interference method verifies the role of these new molecular targets in the treatment of PD.

**Results:**

We demonstrated that a 10-day rTMS treatment on the M1 motor cortex significantly improved motor dysfunction in PD patients. The beneficial effects persisted for up to 40 days, and were associated with an increase in peripheral Tregs. There was a positive correlation between Tregs and motor improvements in PD cases. Similarly, a 10-day rTMS treatment on the brains of MPTP-induced PD mice significantly ameliorated motor symptoms. rTMS reversed the downregulation of circulating Tregs and tyrosine hydroxylase neurons in these mice. It also increased anti-inflammatory mediators, deactivated microglia, and decreased inflammatory cytokines. These effects were blocked by administration of a Treg inhibitor anti-CD25 antibody in MPTP-induced PD mice. Quantitative proteomic analysis identified TLR4, TH, Slc6a3 and especially Syt6 as the hub node proteins related to Tregs and rTMS therapy. Lastly, we validated the role of Treg and rTMS-related protein syt6 in MPTP mice using the virus interference method.

**Conclusions:**

Our clinical and experimental studies suggest that rTMS improves motor function by modulating the function of Tregs and suppressing toxic neuroinflammation. Hub node proteins (especially Syt6) may be potential therapeutic targets.

**Trial registration:**

Chinese ClinicalTrials, ChiCTR2100051140. Registered 15 December 2021, https://www.chictr.org.cn/bin/project/edit?pid=133691

**Graphical Abstract:**

rTMS is a safe and non-invasive method for Parkinson's disease. In this study, we showed the proportion of CD4+CD25+CD127- regulatory T-cells (Tregs) in the peripheral blood was significantly increased after rTMS treatment. Similar effects of rTMS treatment were verified in MPTP-induced PD mice. Proteomic analysis and RNA interference analyses identified TLR4, TH, Slc6a3 and especially Syt6 as hub node proteins that can be modulated by rTMS therapy in PD.

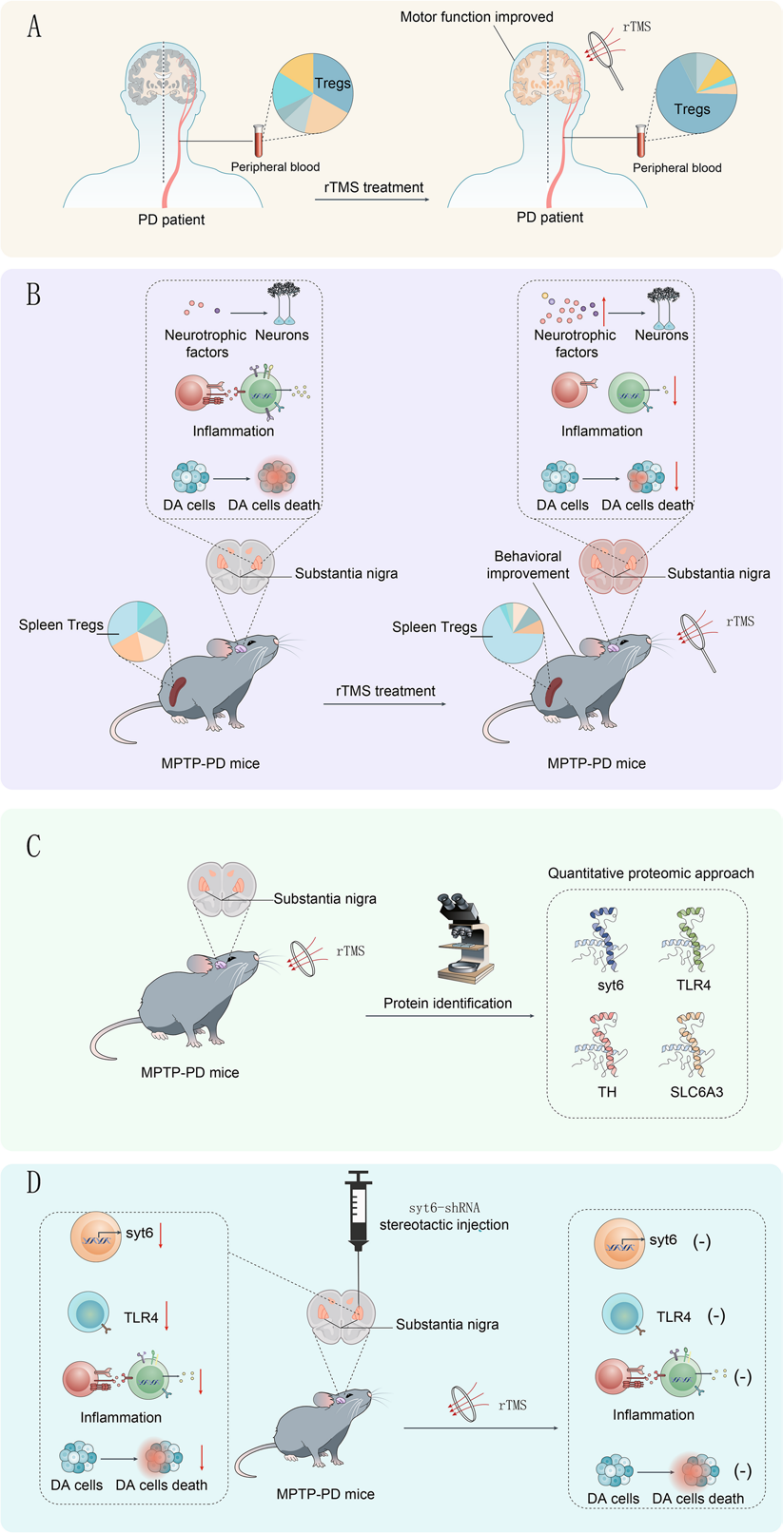

**Supplementary Information:**

The online version contains supplementary material available at 10.1186/s13024-024-00770-4.

## Background

Parkinson’s disease (PD) is a progressive neurodegenerative disorder in which Dopamine (DA) neurons degenerate and neuroinflammatory responses play crucial roles in its pathogenesis [1–4]. There is increasing evidence that the abnormal activation of peripheral immune cells and circulating mediators, including microglia and T cells, actively contribute to neurodegeneration and disease progression in PD [5–9]. Microglia are the most abundant resident immune cells in the brain, and these cells are activated in response to chronic inflammation and produce proinflammatory cytokines in PD [3, 10].

In the immune system, regulatory T cells (Tregs) are an immunosuppressive T cell subset that are important regulators of neuroinflammatory tolerance and preserve immune homeostasis in the central nervous system (CNS) and peripheral circulation [11–14]. Recent studies have shown that Tregs exert neuroprotective effects on some neurological diseases, such as amyotrophic lateral sclerosis (ALS) and ischemic stroke, possibly by mediating the suppression of toxic neuroinflammation and regulating neurotrophic factors in the CNS and peripheral circulation [15–18]. It has been shown that an increase in Tregs in PD models significantly prevents dopaminergic neuronal loss and behavioral changes, and attenuates inflammatory reactions in the CNS [11, 13, 19]. One recent study by Park et al., also suggest that co-transplanting autologous Treg cells could effectively provide a potential strategy to achieve better clinical outcomes for cell therapy in Parkinson's disease [20]. Hence, the role of Tregs in PD needs to be further investigated.

Repetitive transcranial magnetic stimulation (rTMS) is a safe and noninvasive neuromodulation technique that has been used to improve motor symptoms in PD [21–24]. However, the reported outcomes of rTMS in PD have been inconsistent [22]. A recent study has suggested that multi-session of high-frequency rTMS over the primary motor cortex (M1, especially bilateral M1) with a total of 18, 000–20, 000 pulses may be optimal for motor improvement in PD [25]. However, the molecular mechanisms underpinning the therapeutic effects of rTMS on PD have not been elucidated. Animal studies have shown that rTMS can play a neuroprotective role in PD animal models through anti-apoptosis and anti-inflammation [26, 27]. There is a suggestion that proinflammatory cytokines (IFNγ and IL-17A) in the peripheral blood of PD patients may change after TMS treatment [28]. A decrease in the proportion of Tregs in the peripheral blood of PD may be an important feature of the peripheral immune response in PD [13, 29, 30]**.** Therefore, we hypothesize that rTMS may exert its modulatory effects on Tregs in PD.

To test this hypothesis and address current gaps in knowledge, we first designed a prospective clinical trial in PD patients to evaluate the effect of rTMS on motor dysfunction and investigated the relationship between rTMS and changes in circulating Tregs. We next validated the clinical findings using an experimental MPTP mouse model. In addition, we also investigated the mechanisms of Tregs in regulating the therapeutic effects of rTMS. Last, we screened and identified Treg-related hub node proteins in the MPTP model.

## Materials and methods

### Ethics statement

The human studies were approved by the Ethics Committee of Zhujiang Hospital of Southern Medical University, and all patients provided written informed consent prior to recruitment into the study and was conducted in accordance with the principles of the Helsinki Declaration revised in 1975 and the National Institutes of Health Policy and Guidelines for Human Subjects issued in 1999. All mouse experiments were approved by the Experimental Animal Ethics Committee of Zhujiang Hospital of Southern Medical University.

### Clinical study

From Sep 2021 to Jan 2022, 60 PD patients were prospectively recruited in a randomized, double-blinded study (the main researchers, raters, experienced neurologists and patients involved in the study were not aware of the rTMS treatment protocol, except the therapists who did rTMS treatment for PD) from Zhujiang Hospital (Trial Registration Chinese ClinicalTrials. gov Identifier: ChiCTR2100051140). All participants provided written consent to participate in the investigation and allowed investigators to examine their blood samples. Informed written consent was obtained from patients and family members. The enrolled PD patients were interviewed by two experienced neurologists. Characteristics of PD patients, clinical ratings and neuropsychological tests were performed in Table 1. The exclusion Criteria include: 1.Patients with secondary Parkinson's syndrome or Parkinson's overlap syndrome caused by vascular factors, toxins, medications, etc.; 2. PD patients with persistent head tremors; 3. History of Deep Brain Stimulation (DBS) or ablative surgery in the past;4. Other contraindications for rTMS, such as history of epileptic seizures, pregnant women, individuals with cardiac pacemakers, those with metal implants in the body, intracranial hypertension, or severe bleeding tendencies;5. Individuals who have received rTMS treatment within the last 6 months;6. As assessed by the researchers, other factors that make the patient unsuitable for participation in the study or those who are unable to commit to completing follow-up visits. All participants with PD received an EEG before inclusion and fulfilled the Movement Disorder Society Clinical Diagnostic Criteria for Idiopathic PD [31] and underwent extensive clinical examinations. 54 PD patients were ultimately recruited (6 PD patients were ultimately excluded due to four individuals elected not to partake in the study and two subjects were identified as having secondary parkinsonism) and received rTMS treatment or sham rTMS treatment according to random numbers. Random numbers were performed as follows. Computer SAS software was used to randomly group according to a single center, generate random numbers, and the treatment scheme is hidden using opaque envelopes. The envelopes are kept by the treatment provider and opened and sealed by the treatment provider in the order of the patient's visit. Unless the conditions for unblinding are met, the treatment plan cannot be disclosed under any circumstances. The follow-up examinations were done by the same rater and under the same time and all the patients were ON-condition. Sample size was based on a calculation of the results of pre-experiments. The rTMS were delivered by MagPro R30 (Tonica Elektronik A/S, Denmark). The specific rTMS treatment parameters are as follows: initially, high-frequency rTMS (100% resting motor threshold, 10 Hz, 1000 pulses) is administered to the primary motor cortex of the left hemisphere. Subsequently, after a 5-min treatment interval, high-frequency rTMS (100% resting motor threshold, 10 Hz, 1000 pulses) is applied to the primary motor cortex of the right hemisphere. This bilateral hemispheric treatment is conducted daily. The treatment protocol spans over two weeks, with a consecutive 10 working days of treatment for each hemisphere. During the 2-week rTMS treatment period, the dosage of the original treatment drug remained unchanged. The sham rTMS treatment protocol was the same as the rTMS treatment protocol, except that a Cool-B65 P CO coil was used for the sham treatment, while a Cool-B65 A CO coil was used for the rTMS treatment. The alternative sham stimulation was also proceeded for two-week. At the start of each treatment, the resting motor threshold (RMT) of the patient's cortex was measured according to relative frequency method [32]. Specifically, the patients were seated in an armchair with a silversilver chloride surface electrode placed over the abductor pollicis brevis muscle contralateral to each hemisphere. The hot spot was determined using the MagPro R30 Stimulator TMS System (Tonica Elektronik A/S, Denmark) and octagonal coil. The octagonal coil was placed over the scalp and repositioned until the maximal motor evoked potential (MEP) was elicited. After determining the hot spot, the RMT was obtained by delivering single pulse transcranial magnetic stimulation to the hot spot. The RMT was defined as the lowest TMS intensity capable of eliciting a MEP greater than a 50 μV peak-to-peak amplitude in five of the ten subsequent trials. CD4 + CD25 + CD127- cells, CD4 + CD25(low)CD45RA + [naive Treg (nTreg) cells] and CD4 + CD25(high)CD45RA- [activated Treg (aTreg) cells] subsets in the peripheral blood were assessed by flow cytometry and IFN-γ, TNF-α, IL-17α, IL4, IL10, TGF-β1 in the peripheral blood were assessed by Enzyme-linked immunosorbent assay (ELISA) prior to treatment and day 13. Hoehn and Yahr and MDS-UPDRS III were performed prior to treatment and on Days 13, 19, and 40 following the culmination of the two-week rTMS therapy.

**Table 1:** Baseline characteristics of PD patients in sham and rTMS group

Characteristics	Sham	rTMS	*P* value
			Sham vs rTMS
n	27	27	
Gender (M/F)	14/13	14/13	> 0.9999
Age (years)	63.48 ± 9.57	63.26 ± 8.17	0.9273
MDS-UPDRS III (score)	51.33 ± 11.68	50.67 ± 10.47	0.8260
20–30 (n)	1	1	
30–50 (n)	10	9	
> 50 (n)	16	17	
H & Y scale	2.61 ± 0.68	2.67 ± 0.65	0.7610
1(n)	0	1	
1.5(n)	6	2	
2(n)	1	4	
2.5(n)	3	2	
3(n)	16	17	
4(n)	1	1	
LED (mg/day)	838.0 ± 484.0	830.8 ± 412.5	0.9534
A(n)	2	2	
F(n)	1	2	
A + C(n)	0	1	
A + D(n)	2	2	
A + E(n)	1	1	
A + B + C(n)	1	1	
A + B + D(n)	1	0	
A + C + D(n)	4	4	
A + C + E(n)	2	3	
A + C + G(n)	1	2	
A + D + E(n)	2	1	
A + D + F(n)	1	0	
A + B + C + D(n)	3	2	
A + B + C + E(n)	1	0	
A + C + D + E(n)	1	1	
A + C + D + F(n)	1	1	
A + C + E + G(n)	1	1	
A + D + E + G(n)	0	1	
A + B + C + D + E(n)	1	1	
A + C + D + E + G(n)	1	1	
Duration since symptom onset(month)	71.26 ± 46.54	86.81 ± 69.53	0.3385
Motor subtype			> 0.9999
Mixed type	10	11	
Tremor type	12	10	
akinetic-rigid type	5	6	
The presence of motor fluctuations(Yes/No)	10/17	8/19	0.5723

LED refers to the equivalent dose of levodopa, A = Dobassrazide tablets, B = Carlevodopa controlled-release tablets, C = Entacapone, D = Pramipexole hydrochloride, E = Amantadine hydrochloride, F = Rasagiline, G = Ropinirole hydrochloride sustained-release tablets. A indicates the use of dobasilazine tablets as monotherapy; F indicates the use of rasagiline monotherapy; A + D indicates the combined use of dobasilazine tablets and pramipexole hydrochloride; the other combination drugs can be deduced by analogy. Data are means ± SD unless otherwise indicated

The peripheral blood was obtained from PD patients [33] and lysed in ACK lysis buffer (Biosharp, China).CD4 T cells were then purified using magnetic bead separation (Miltenyi Biotec GmbH, Bergisch Gladbach, Germany). The cells were stained with anti-CD25 and anti-CD127 (all from BD Biosciences) for analysis by flow cytometry. Following the acquisition of sample data on a FACSCalibur flow cytometer (BD Biosciences), the results were generated in graphical and tabular formats using FlowJo V10 software (TreeStar Inc., Ashland, USA).

### Animal study

Based on our clinical findings of an association between rTMS and changes in circulating Tregs of PD patients, we next conducted studies in a PD mouse model to validate the results. We determined whether the Tregs in the spleen of the PD mouse model changed after rTMS treatment and investigated the potential mechanisms that led to these changes. We applied a similar rTMS treatment protocol in MPTP mice. Male C57BL/6 mice (8 weeks old, 22–24 g) received intraperitoneal (i.p.) injection of 1-methyl-4-phenyl-1, 2, 3, 6-tetrahydropyridine (MPTP) (Sigma–Aldrich, USA) (30 mg/kg) for 5 days as PD animal model, while control mice received the same dose of normal saline. Another group of MPTP mice received i. p. injection of anti-CD25 monoclonal antibody (1 mg/kg; Thermo, USA) for three consecutive days to block Tregs in the spleen, to investigate whether the therapeutic effect of rTMS on MPTP mice is related to changes in Treg level in the spleen. In addition, we also evaluated the behavior of mice by pole climbing test and detected the expression of dopamine cells, neurotrophic factors BDNF, GDNF, microglia and inflammatory factors in the SN of mice. We next screened the hub node proteins related to rTMS on MPTP mice by quantitative proteomics analysis with TMT labeled of whole proteins. Finally, to better understand the role of hub proteins in MPTP mice, RNA interference (RNAi) mediated Adeno-associated virus (AAV) vector was injected into midbrain via a stereotactic midbrain approach to block hub proteins level in SN. Immunofluorescent staining and western blots (WB) were used to detect the transfection efficiency. The changes of hub protein levels in SN were detected by immunofluorescence staining and the inflammatory cytokines in the ventral midbrain were detected by ELISA after the hub proteins were interfered by the virus.

For the sample size in animal experiments, a power analysis was performed. A sample size of at least *n* = 8 per group was determined to reach a statistical significance of 0.05, in detecting an effect size of at least 1.06 with 80% power. Mice were assigned randomly to the experimental groups. The investigators were blinded to the identities of treatment groups.

### Animals and MPTP-PD model

Male C57BL/6 mice (8 weeks old, 22–24 g) were obtained from Liaoning Changsheng Biotechnology Co., Ltd. (Liaoning, China). Before the experiment, all mice were housed at 20–22 °C with a 12 h: 12 h light/dark cycle and food and water provided ad libitum for 7 days. The CD25 receptor is a key site for activating Treg, and as the hypothesis is that rTMS may exert its modulatory effects on Treg in PD, we used anti CD25 injections to specifically block Treg to determine if rTMS still has an impact on PD. A total of 189 male mice were randomly divided into 9 groups as follows: (1) the saline normal control group (NC group, *n* = 21 mice) was only administered saline by intraperitoneal (i. p.) injection for 5 days; (2) the NC + sham rTMS group (NC + sham group, *n* = 21 mice), which were treated with i. p. injections of saline (30 mg/kg) for 5 days followed by sham rTMS treatment; (3) the NC + rTMS (10 Hz) group (*n* = 21 mice), which were administered i. p. doses of saline (30 mg/kg) for 5 days before receiving rTMS (10 Hz) therapy; (4) the MPTP group (*n* = 21 mice), which received i. p. injections of 1-methyl-4-phenyl-1, 2, 3, 6-tetrahydropyridine (MPTP) (Sigma–Aldrich, USA) (30 mg/kg) for 5 days; (5) the MPTP + sham rTMS group (*n* = 21 mice), which were treated with i. p. injections of MPTP (30 mg/kg) for 5 days followed by sham rTMS treatment; (6) MPTP + rTMS (10 Hz) group (*n* = 21 mice), which were administered i. p. doses of MPTP (30 mg/kg) for 5 days before receiving rTMS (10 Hz) therapy; (7) the MPTP + block group (*n* = 21 mice), which received 3 days of continuous i. p. injections of anti-CD25 monoclonal antibodies (1 mg/kg) followed by 5 days of i. p. injections of MPTP (30 mg/kg); (8) the MPTP + block + sham (10 Hz) group (*n* = 21 mice), which was administered i. p. injections of anti-CD25 monoclonal antibodies (1 mg/kg, Thermo, USA) for 3 days before receiving i. p. injections of MPTP (30 mg/kg) for 5 days before receiving shamtherapy; and (9) the MPTP + block + rTMS (10 Hz) group (*n* = 21 mice), which was administered i. p. injections of anti-CD25 monoclonal antibodies (1 mg/kg, Thermo, USA) for 3 days before receiving i. p. injections of MPTP (30 mg/kg) for 5 days before receiving sham therapy.

### rTMS treatment

A transcranial magnetic stimulator (MagVenture A/S, Denmark) connected to a circular coil (Cool-40 Rat Coil) with a diameter of 40 mm was used to deliver rTMS. the rTMS treatment regimen for mice is consistent with the treatment regimen for PD patients. In the sham rTMS group, the stimulus was delivered by fixing the coil 10 cm above the heads of the mice to ensure that the mice felt the clicking sound or vibrations generated by the rTMS coil but did not receive brain stimulation. To minimize the discomfort of the mice, their movements were carried out in a soft plastic funnel, therefore, on the first day, they were restrained without stimulation for 10 s to familiarize them with the experimental procedure. At the start of each treatment, the resting motor threshold was measured as previously described [34]. Briefly, a standard electromyographic (EMG) machine (Counterpoint, Dantec Medical Inc., Denmark) was used for recording of the motor evoked potentials (MEPs). Sampling frequency was 51.4 kHz, high and low pass filter settings were 10 and 3000 Hz. A 0.5 mm bipolar EMG needle (DANTEC) placed in the right hindlimb biceps femoris muscle with a ground electrode 10 mm proximal to the recording electrode was used for recording the muscle activity. The motor threshold was defined as a reproducible motor evoked potential in five consecutive stimuli with an interstimulus interval > 3 s and an amplitude > 50 mV. The real and sham rTMS treatments did not induce seizures or other behavioral changes during the treatment period.

### Pole climbing test

We assessed motor dysfunction by performing pole tests [35] Briefly, the mice were placed head-up on the top of the pole (height 50 cm, diameter 1 cm), and their return to the bottom was timed. Timing began when the mouse was released and stopped when one hindlimb reached the bottom. The test was repeated 3 times for each mouse after they were trained once at 30 min intervals, and the average time was used for data analysis. The raters were blinded for the treatment group.

### Flow cytometric analysis of CD4 + CD25 + Foxp3 + Tregs in the spleen

The spleens of male C57BL/6 J mice [19] were removed and homogenized by a tissue grinder and a wire mesh screen. Red blood cells were then lysed in ACK lysis buffer. Then CD4 + T cells were then purified using magnetic bead separation (Miltenyi Biotec GmbH, Bergisch Gladbach, Germany). The cells were stained with anti-CD25 and anti-Foxp3 (BD Biosciences, USA, 567,456) for flow cytometric analysis. Following the acquisition of sample data on a FACSCalibur flow cytometer (BD Biosciences), the results were generated in graphical and tabular formats using FlowJo V10 software (TreeStar Inc., Ashland, USA).

### Immunohistochemical staining

The mice were anesthetized with sodium pentobarbital, sacrificed, and transcardially perfused with normal saline containing 0.5% sodium nitrate and heparin (10 U/ml) followed by 4% paraformaldehyde (PFA) dissolved in 0.1 M phosphate buffer. The brain was dissected and postfixed in 4% PFA at 4 °C for 18–24 h and then immersed in 20% sucrose at 4 °C until the brain dropped to the bottom, followed by immersion in 30% sucrose at 4 °C for 24 h. The brains were embedded in optimal cutting temperature compound (O. C. T. Compound, Tissue-Tek, USA.) We prepared coronal sections of the frozen brains with a cryostat microtome (CM1950, Leica, Germany). Frozen brains were sectioned into 40-µm-thick coronal sections. All sections were collected and processed for immunohistochemical staining. For antigen retrieval, the tissue slices were submerged in 0.01 M sodium citrate buffer (pH 6.0) and rinsed twice in PBS. The tissue slices were then treated for 1 h at 37 °C in PBS containing 0.2% v/v Triton X-100, 0.02% w/v sodium azide, and 5% v/v goat serum. Primary antibodies against tyrosine hydroxylase TH (1: 800, Proteintech, USA, 25,859–1-AP), BDNF (1: 500, Abcam, England, ab108319), GDNF (1: 500, Abcam, England, ab18956) and Iba-1 (1: 500, Abcam, England, ab178846) SYT6 (1: 250, SANTA CRUZ, sc390321), TLR4 (1: 250, Invitrogen, 710,185) were added to the slices and incubated overnight at 4 °C. Then, the sections were treated with the appropriate biotinylated secondary antibody, followed by Vectastain ABC reagent (Vector Laboratories, Burlingame, CA, USA) and 3, 3’-diaminobenzidine (Sigma–Aldrich, Vienna, Austria). The stained slices were dehydrated and coverslipped with Entellan before being mounted on slides (Merck, Darmstadt, Germany). IgG conjugated with Alexa 594 (1: 500, Abcam, England, ab150080) was used for immunofluorescent staining. Mounting medium was used to cover the sections (Dianova, Hamburg Germany). The numbers of TH + , SYT6, TLR4 in the substantia nigra pars compacta (SNc) were estimated using unbiased stereological methods (Stereo Investigator, MicroBrightField, VT). The numbers of activated Iba-1-positivemicroglia in the SNc were estimated using the optical fractionator probe with the stereologist. Microglia were classified as activated if the cell body was visibly increased in diameter and the cell had shortened and thickened processes. The three stereologists were blinded to the treatment received. Additionally, we determined the average optical density value of BDNF and GDNF immunoreactivity through densitometry, employing the freely available ImageJ software (National Institutes of Health, Bethesda, MD, USA). All histochemical data statistics was conducted in a single-blind manner by four experimenters. FX renumbers each group of fluorescent films in an experiment and randomly divides them into three groups (random number method) and assigns them to three investigators (YQL, ZCX and Q.W). Each film takes 5 fields of view for statistical averaging, and finally the data is summarized with FX.

### Quantitative proteomics technology with TMT labeling of whole protein

Twelve brain tissue from 4 groups (saline normal control, MPTP, MPTP + rTMS, MPTP + block + rTMS) were collected for proteome Tandem Mass Tag (TMT) analysis. The collected samples were processed for TMT analysis in JingJie Bio-tech. Output was visualized using R and CummeRbund. Heatmaps were generated in R using pHeatmap and R ColorBrewer. Result was analyzed according to Maxquant (v1.6.15.0) database.

### Molecular docking method

The protein data bank (PDB) database (https://www.rcsb.org/) was used to obtain the PDB file which has the active structure of the target protein, The smaller Resolution value and complete protein binding pockets were used as the screening conditions to select the crystal structure for subsequent analysis. Refer to the previous literature [36–40], according to the amino acid sequence provided by Unirpot, SWISS-MODEL was used (https://swissmodel.expasy.org/interactive) to perform online homology modeling of proteins without ready-made protein crystal structures in the PDB database. Select the result with homology ≥ 30%, and then according to the scoring screen to evaluate the model quality with GMQE and QMEAN values when the homology reaches the standard, the template with larger GMQE and QMEAN close to 0 was chose. Then, online protein–protein docking is performed through Z-dock (https://zdock.umassmed.edu), the version is selected as V3.0.2 [41, 42], and the docking result image uses PyMol 2. 3.5 for image rendering.

### Path enrichment analysis and PPI network construction

GO database (http://geneontology.org/) was used for pathway enrichment analysis [43], obtain immune-related pathway genes, and String database (https://www.string-db.org/) was used to construct protein online Protein interaction network (PPI network) [44], the species is selected as Mus musculus, the minimum required interaction score is set to 0.4, and the other conditions are the default settings.

### Western blotting

Tissues were lysed in a detergent-based lysis buffer (50 mM Tris, pH 7.4, 150 mM NaCl, 1% Triton X-100, 1% sodium deoxycholate, 0.1% SDS and 1 mM PMSF). Samples were then centrifuged for 10 min at 13, 000 g at 4 °C, and the supernatant was collected and quantified with the BCA Protein Assay Kit (Thermo Scientific, USA). Equal amounts of protein sample were separated by 10% SDS-PAGE. Antibodies used for western blotting were: Syt6 (1: 1000, abcam, USA), TLR4 (1: 1000, abcam, USA), TH (1: 1000, proteintech, USA), Slc6a3 (1: 1000, abcam, USA) and β-actin (1: 10,000, Thermo, USA). Antibody binding was detected using the secondary antibodies (Peroxidase-Conjugated AffiniPure Goat Anti-Rabbit/Mouse IgG (H + L), 1: 5000, thermofisher, USA). All samples were normalized to β-actin. All quantification was calculated with Alpha Ease FC software (Alpha Innotech Corporation, USA).

### ELISA assay

Cytokine levels of IFN-γ, TNF-α, IL-17α, IL4, IL10 and TGF-β1 in the serum of PD patients were measured using commercial ELISA kits (IFN-γ ELISA kit (Abcam, England, ab46025), TNF-α ELISA kit (Abcam, England, ab181421), IL-17α ELISA kit (Abcam, England, ab216167), IL4 ELISA Kit (Abcam, England, ab215089), IL-10 ELISA kit (Abcam, England, ab100549), TGF-β1 ELISA kit (Sigma–Aldrich, USA,RAB0460) in accordance with the manufacturer’s protocols. Brain tissue was collected from the different groups of mice, and then the tissue from the ventral midbrain was separated and homogenized with lysis buffer on ice. The supernatant was collected at 12, 000 rpm at 4 °C and centrifuged for 10 min for subsequent experiments. The proinflammatory cytokines IL-6, IFN-γ, TNF-α, IL-1β, IL-10 and TGF-β1 were measured using commercial ELISA kits (IL-6 ELISA kit (Abcam, England, ab100713), IFN-γ ELISA kit (Abcam, England, ab100690), TNF-α ELISA kit (Abcam, England, ab229393), IL-1β ELISA Kit (Sigma–Aldrich, USA, RAB0275), IL-10 ELISA kit (Abcam, England, ab100697), TGF-β1 ELISA kit (Abcam, England, ab119557) in accordance with the manufacturer’s protocols.

### Virus constructs and stereotaxic surgery

The scramble short hairpin RNA (shRNA) (5’-CCTAAGGTTAAGTCGCCCTCG-3’) and shRNA coding sequences targeting mous syt6 (5’-ATGAAAGCGAGACGCTGATTG-3’), (5’- ATGTCTCCAGCGTGGACTATG-3’) and (5’- GCGGAAGTTCTGACCCTTA-3’) were cloned into the PAAV-U6-shRNA(Syt6)-CMV-EGFP-WPRE vector (Obio Technology, Shanghai, China). Adeno-associated viruses (AAVs) described above were packaged by Obio Technology into serotype 2/9.After mice were anesthetized and placed in the stereotaxic frame, 2 μl of purified and concentrated AAV(10^12^ IU/mL) was unilateral injected into the right SNc (AP =  − 3.2 mm; ML =  ± 1.2 mm, DV =  − 4.6 mm) at a rate of 0.2 μl/min according to previously described protocols [45]. The 5-μl Hamilton syringe was kept in place for 10 min before being slowly retracted within 5 min.

### Statistical analysis

Statistical analyses were performed using GraphPad Prism 8.0 software. Data with a normal distribution are presented as the mean ± SD. Intra-group comparisons of squared differences were conducted using paired sample t-tests, while inter-group comparisons were made using two independent samples t-tests. Data that did not conform to a normal distribution are presented with comparisons made using non-parametric tests. Counting data are presented as frequencies, with comparisons made using the χ2 test. A two-sided test was chosen, and a P-value less than 0.05 was considered statistically significant. Correlation between the change of Treg ratio in peripheral blood and the change of MDS-UPDRSIII before and after treatment with rTMS were calculated by the Pearson correlation. After flowcytometry, CD4 subsets were Determined as a percentage of all lymphocytes gated using forward and side scatter, and as a percentage of all leukocytes bydual-platform analysis using full blood cell counts from the same blood collection. Mouse flow cytometry data, total times of pole climbing test, TH, BDNF, GDNF, Syt6, TLR4 immunohistochemistry, IL-6, IFN-γ, TNF-α, IL-1β, IL-10 and TGF-β1 enzyme-linked immunosorbent assay and Syt6, TLR4, Slc6a3 and TH western blotting were analyzed using One-way ANOVA followed by the least significant difference (LSD) for post hoc comparisons. The data are representative of at least three independent experiments.

## Results

### rTMS increased the proportion of Tregs, attenuated inflammation and improved motor impairment in PD patients

**Fig. 1:**
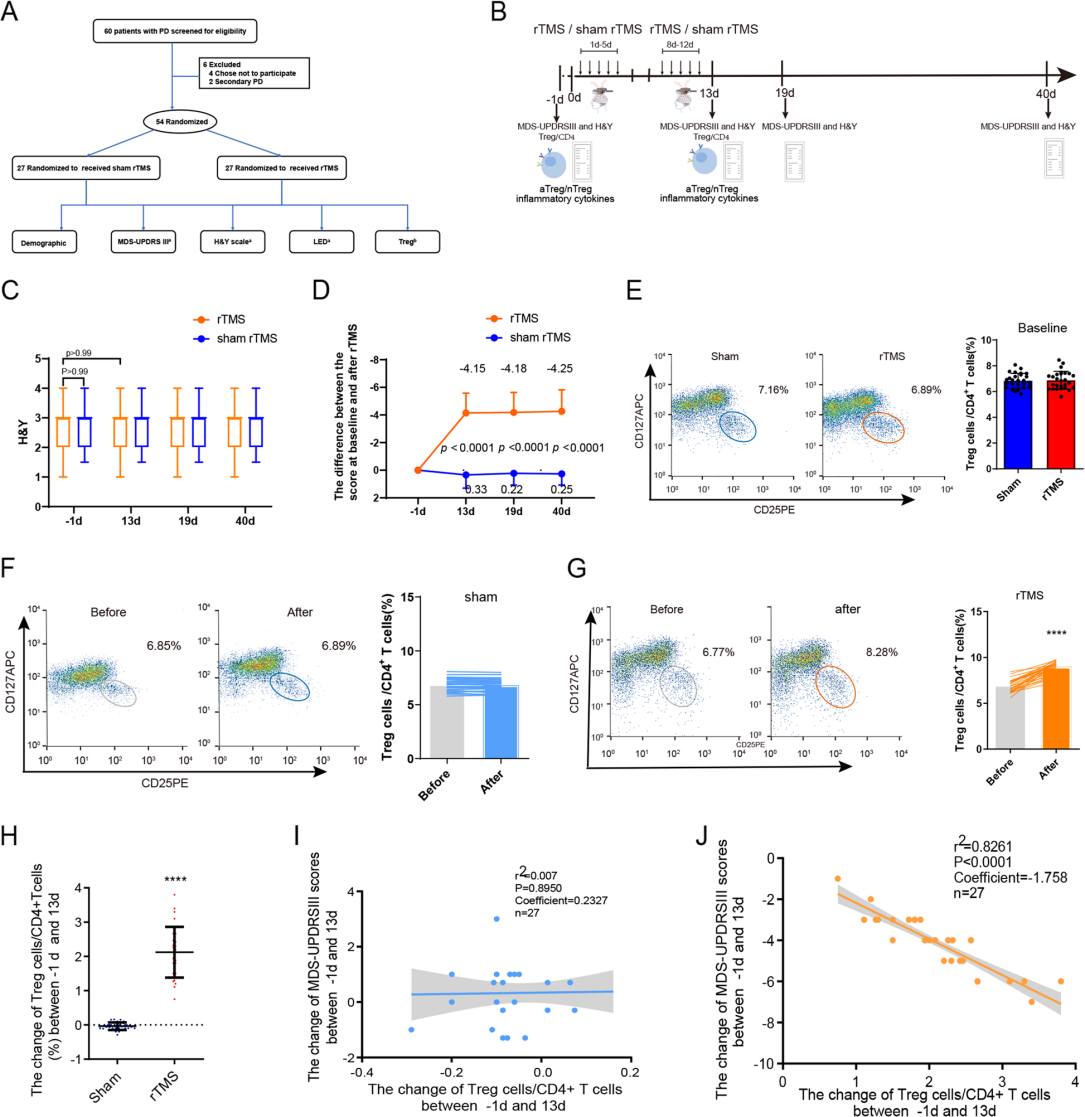
rTMS treatment improved motor functions and increased the proportion of peripheral Tregs in PD patients. **A** Study flow chart. Sixty patients with PD were screened for eligibility in this clinical study between Sep 2021 and Jan 2022. A total of 6 PD patients were ultimately excluded. Finally, the 54 PD patients were randomized (1: 1) to divided into sham and rTMS group. Demographic data, basic examinations including electroencephalography, medication dosage before and after rTMS or sham treatment were carried out in both groups of patients. Tregs, aTreg, nTreg, proportion and the levels of IFN-γ, TNF-α, IL-17α, IL4, IL10 and TGF-β1 in the peripheral blood were measured, H&Y and MDS-UPDRS III scores were performed at the same time point before and after rTMS or sham treatment. **B** Tregs, and aTreg/nTreg proportion in the peripheral blood were assessed by flow cytometry prior to treatment and 1 day after the treatment. Hoehn and Yahr and MDS-UPDRS III were performed prior to treatment and on Days 13, 19, and 40 after the treatment. **C** No significant difference between the Mean H & Y ± SD on Days -1, 13, 19 and 40 in the sham and rTMS groups. (sham vs. rTMS groups on days -1: *n* = 27, *P* = 0.761, t = -0.306, d. f. = 52; sham vs. rTMS groups on days 13: *n* = 27, *P* = 0.761, t = -0.306, d. f. = 52; sham vs. rTMS groups on days 19: *n* = 27, *P* = 0.761, t = -0.306, d. f. = 52; sham vs. rTMS groups on days 40: *n* = 27, *P* = 0.761, t = -0.306, d. f. = 52). **D** There was a significant difference in the difference between the MDS-UPDRS III scores of the rTMS group and the sham group on day 13, 19, and 40 and their respective MDS-UPDRSIII scores on day -1.(rTMS vs. sham groups between days 13 and -1: *n* = 27, *P* < 0.0001, t = 15.64, d. f. = 208; rTMS vs. sham groups on 19 and -1: *n* = 27, *P* < 0.0001, t = 15.38, d. f. = 208; rTMS vs. sham groups on on 40 and -1: *n* = 27, *P* < 0.0001, t = 15.77, d. f. = 208). **E** The baseline of the proportion of CD4 + CD25 + CD127- Treg cells in the peripheral blood displays no significant difference between sham and rTMS group. (sham vs. rTMS groups: *n* = 27, *P* = 0.796, t = 0.26, d. f. = 52). **F** The peripheral blood CD4 + CD25 + CD127- Treg cells before and after sham rTMS treatment in the sham treatment group shows no significant difference. (before vs. after: n = 27, *P* = 0.185, t = 1.363, d. f. = 26). **G** The peripheral blood CD4 + CD25 + CD127-Treg cells before and after rTMS treatment in the rTMS treatment group shows significant difference. (before vs. after: *n* = 27, *P* < 0.000, t = -14.88, d. f. = 26). **H** Mean change in the proportion of Tregs/CD4 + T cells in the peripheral blood ± SD between days -1 and 13 in the sham and rTMS groups. (*n* = 27, *P* < 0.0001, t = 14.98, d. f. = 52). **I** No correlation between the change in the proportion of Tregs/CD4 + T cells and the change in MDS-UPDRSIII scores (between days -1 and 13) in sham group (*n* = 27, r2 = 0. 0007, *p* = 0.895). **J** Negative correlation between the change in the proportion of Tregs/CD4 + T cells. and the change in MDS-UPDRSIII scores (between days -1 and 13) in rTMS group. (*n* = 27, r2 = 0.8261, *p* < 0.0001). rTMS = repetitive transcranial magnetic stimulation; H & Y = Hoehn-Yahr; MDS UPDRS III = Movement Disorder Society Unified Parkinson's Disease Rating Scale part III; SD = Standard Deviation; Data are presented as mean ± SD; two-tailed unpaired t test (**C**, **D**, **E**, **H**); paired sample t test (**F**, **G**); linear regression analysis (**I**, **J**). ****P < 0.0001. Each data point represents an individual subject. Comparisons with no asterisk had a *P* > 0.05 and were considered not significant

We conducted a clinical trial that included a total of 60 PD patients (6 PD patients were ultimately excluded). Fifty-four PD patients were divided into two groups; one group was treated with 10-day rTMS (10 Hz) in the M1 area, and the other group received sham rTMS treatment (Fig. 1A, B), followed by Hoehn and Yahr and MDS-UPDRS III assessments at Days 1, 13, 19, and 40 (Fig. 1A, B; Table 1). Our clinical trial showed that the MDS-UPDRS Part III score was significantly reduced in PD patients receiving rTMS as compared to the sham control group on the day following the completion of a two-week rTMS treatment protocol (-4.15 ± 1.689, *P* < 0.0001). Furthermore, the MDS-UPDRS Part III score sustained a lower score for up to 40 days post-treatment with rTMS (-4.25 ± 1.716, *P* < 0.0001) (Fig. 1C, D). The baseline clinical and demographic characteristics were not different between the rTMS and sham groups (Table 1). We performed flow cytometry with anti-CD4, CD25 and CD127 antibodies to examine the proportions of CD4 + CD25 + CD127- Treg cells and CD4 + T cells in sham and rTMS groups. The results showed no difference in the proportion of Tregs (% CD4 + T cell population) between the sham and rTMS groups at baseline (Fig. 1E). However, 10-day rTMS treatment (9.0 ± 0.64%) elevated peripheral Treg levels in PD patients (6.87 ± 0.69%) (*p* < 0.0001; Fig. 1G, H), but there was no difference in the peripheral Treg levels in sham group (Fig. 1F). Pearson’s correlation was used to analyze the association between MDS-UPDRS III scores and the proportion of Tregs. We found that the increased proportion of Tregs was negatively correlated with the change in the MDS-UPDRS III scores in the rTMS PD group (*P* < 0.0001; Fig. 1J) but not in the sham treatment group (Fig. 1I). Furthermore, there was an observed increase in the proportion of aTreg/nTreg cells within the peripheral blood (Supplementary Fig. 1A, B, C). Concurrently, levels of IL4, IL10, and TGF-β1 were elevated, while pro-inflammatory markers such as IFN-γ, TNF-α, and IL-17α demonstrated a decrease (Supplementary Fig. 1D, E, F). The MFI of CD25 also shown no difference in the proportion of CD25 between the sham and rTMS groups at baseline (Supplementary Fig. 2A). In addition, the median fluorescence intensity (MFI) of CD25 was increased after the treatment of rTMS compared with sham treatment (Supplementary Fig. 2C).

### rTMS ameliorated behavioral impairment in MPTP-induced mice

**Fig. 2:**
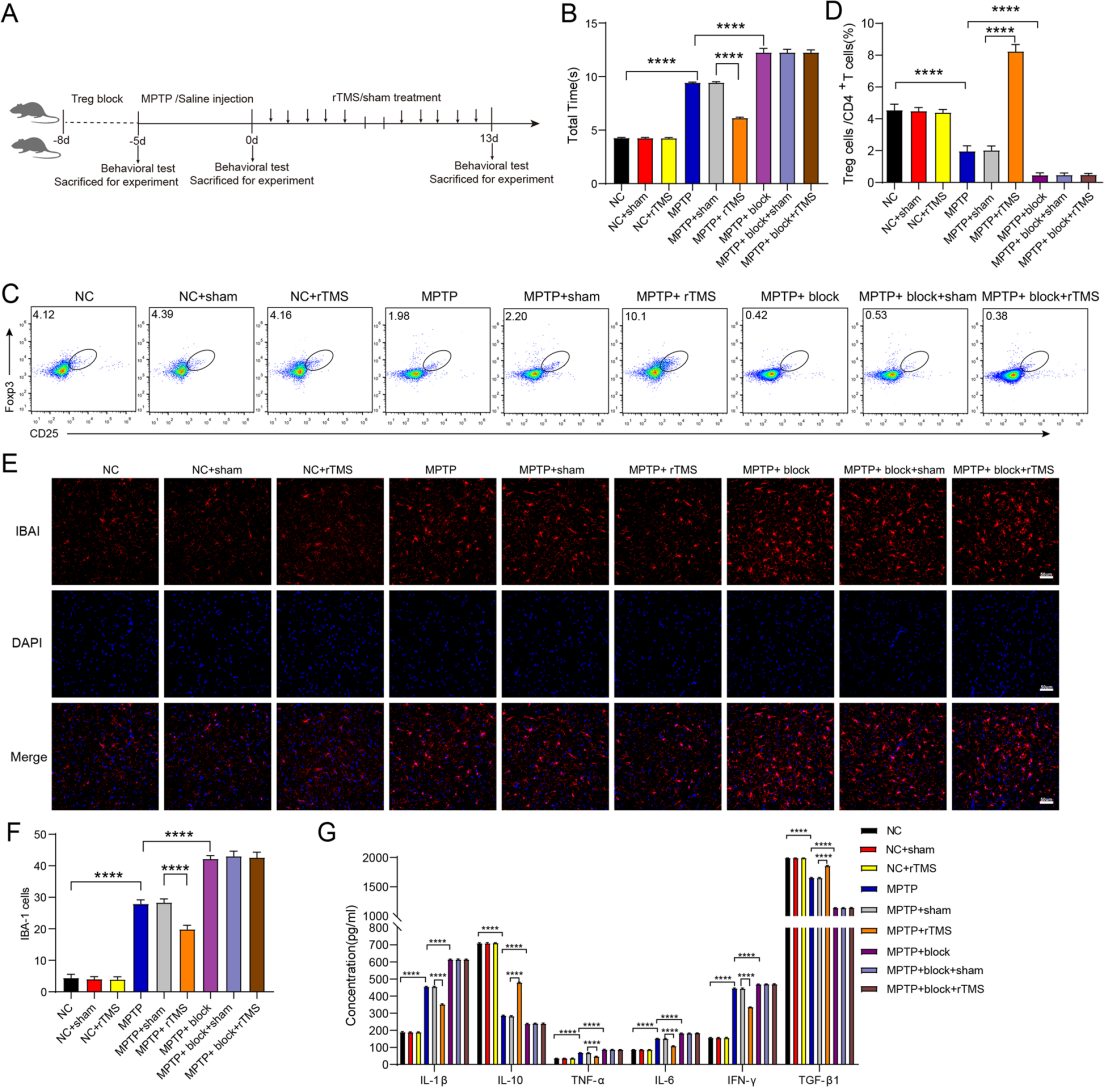
rTMS treatment ameliorated behavioral impairments, increased the proportion of Tregs, suppressed the neuroinflammatory responses in MPTP-induced PD mice, and regulated the potential targets of Tregs. **A** Animal experimental design and rTMS treatment scheme to explore the role and mechanism of rTMS in the MPTP-induced PD mouse model. **B** Quantification of total the times for each group mice that spent in pole climbing test. (F8,99 = 8257, NC vs. NC + sham: *P* > 0.9999; NC vs. MPTP: *P* < 0.0001; NC + sham vs. NC + rTMS: *P* > 0.9999; MPTP vs. MPTP + sham: *P* > 0.9999; MPTP vs. MPTP + rTMS: *P* < 0.0001; MPTP + sham vs. MPTP + rTMS: *P* < 0.0001; MPTP vs. MPTP + block: *P* < 0.0001; MPTP + block + sham vs. MPTP + block + rTMS: *P* = 0.9494, *n* = 12 mice per group). **C** Flow cytometry–based analysis of CD3 + CD4 + CD25 + Foxp3 + Treg cells represen-tative fluorescence-activated cell sorting (FACS) plots of each group of mice. **D** The proportions of CD3 + CD4 + CD25 + Foxp3 + Treg cells/CD4 + T cells in the spleens of mice in each group. (F8, 45 = 608.8, NC vs. NC + sham: *P* > 0.9999; NC vs. MPTP: *P* < 0.0001; NC + sham vs. NC + rTMS: *P* = 0.9998; MPTP vs. MPTP + sham: *P* > 0.9999; MPTP vs. MPTP + rTMS: *P* < 0.0001; MPTP + sham vs. MPTP + rTMS: *P* < 0.0001; MPTP vs. MPTP + block: *P* < 0.0001; MPTP + block + sham vs. MPTP + block + rTMS: *P* > 0.9999, *n* = 6 mice per group). **E** Representative images of microglia in the SN. microglia were visualized by iba-1 staining. Scale bar, 50 μm. **F** Mean microglial immunostaining of Iba-1-positive cells in the SN in each group of mice ± SD. (F8, 81 = 1803, NC vs. NC + sham: *P* = 0.9985; NC vs. MPTP: *P* < 0.0001; NC + sham vs. NC + rTMS: *P* > 0.9999; MPTP vs. MPTP + sham: *P* = 0.9985; MPTP vs. MPTP + rTMS: *P* < 0.0001; MPTP + sham vs. MPTP + rTMS: *P* < 0.0001; MPTP vs. MPTP + block: *P* < 0.0001; MPTP + block + sham vs. MPTP + block + rTMS: *P* = 0.9985, *n* = 10 mice per group). **G** ELISA was used to analyze the protein expression of IL-10, TGF-β1, IL-6, IFN-γ, TNF-α and IL-1β in the ventral midbrain in each group of mice. (IL-1β: F8, 45 = 30,632, NC vs. NC + sham: *P* > 0.9999; NC vs. MPTP: *P* < 0.0001; NC + sham vs. NC + rTMS: *P* > 0.9999; MPTP vs. MPTP + sham: *P* > 0.9999; MPTP vs. MPTP + rTMS: *P* < 0.0001; MPTP + sham vs. MPTP + rTMS: *P* < 0.0001; MPTP vs. MPTP + block: *P* < 0.0001; MPTP + block + sham vs. MPTP + block + rTMS: *P* > 0.9999; IL-10: F8, 45 = 36,052, NC vs. NC + sham: *P* > 0.9999; NC vs. MPTP: *P* < 0.0001; NC + sham vs. NC + rTMS: *P* = 0.9894; MPTP vs. MPTP + sham: *P* = 0.9493; MPTP vs. MPTP + rTMS: *P* < 0.0001; MPTP + sham vs. MPTP + rTMS: *P* <  = 0.0001; MPTP vs. MPTP + block *P* < 0.0001; MPTP + block + sham vs. MPTP + block + rTMS: *P* = 0.9999; TNF-α: F8, 45 = 1767, NC vs. NC + sham: *P* > 0.9999; NC vs. MPTP: *P* < 0.0001; NC + sham vs. NC + rTMS: *P* > 0.9999; MPTP vs. MPTP + sham: *P* = 0.5829; MPTP vs. MPTP + rTMS: *P* < 0.0001; MPTP + sham vs. MPTP + rTMS: *P* < 0.0001; MPTP vs. MPTP + block: *P* < 0.0001; MPTP + block + sham vs. MPTP + block + rTMS: *P* > 0.9999; IL-6: F8, 45 = 7443, NC vs. NC + sham: *P* = 0.9987; NC vs. MPTP: *P* < 0.0001; NC + sham vs. NC + rTMS: *P* > 0.9999; MPTP vs. MPTP + sham: *P* = 0.7244; MPTP vs. MPTP + rTMS: *P* < 0.0001; MPTP + sham vs. MPTP + rTMS: *P* < 0.0001; MPTP vs. MPTP + block: *P* < 0.0001; MPTP + block + sham vs. MPTP + block + rTMS: *P* = 0.6587; IFN-γ: F8, 45 = 25,766, NC vs. NC + sham: *P* > 0.9999; NC vs. MPTP: *P* < 0.0001; NC + sham vs. NC + rTMS: *P* > 0.9999; MPTP vs. MPTP + sham: *P* = 0.9798; MPTP vs. MPTP + rTMS: *P* < 0.0001; MPTP + sham vs. MPTP + rTMS: *P* < 0.0001; MPTP vs. MPTP + block: *P* < 0.0001; MPTP + block + sham vs. MPTP + block + rTMS: *P* > 0.9999; TGF-β1: F8, 45 = 22,257, NC vs. NC + sham: *P *> 0.9999; NC vs. MPTP: *P* < 0.0001; NC + sham vs. NC + rTMS: *P* > 0.9999; MPTP vs. MPTP + sham: *P* > 0.9999; MPTP vs. MPTP + rTMS: *P* < 0.0001; MPTP + sham vs. MPTP + rTMS: *P* < 0.0001; MPTP vs. MPTP + block: *P* < 0.0001; MPTP + block + sham vs. MPTP + block + rTMS: *P* = 0.9998; *n* = 6 mice per group). SN = substantia nigra; LSD = least significant difference; MPTP = 1-methyl-4-phenyl-1, 2, 3, 6-tetrahydropyridine; NC = normal control; Data are presented as mean ± SD; One-way ANOVA with Fisher’s LSD multiple comparison post hoc test (**B**, **D**, **E**, **G**). ****P < 0.0001. Each data point represents an individual mouse. Comparisons with no asterisk had a *P* > 0.05 and were considered not significant

We induced PD-like symptoms and analyzed the impact of rTMS on motor function in MPTP mice. Based on the results of the pole climbing test, MPTP significantly impaired motor function, and rTMS ameliorated this impairment (Fig. 2A, B). Compared to saline normal control mice, MPTP-induced mice took 121.14% more time to turn around and climb down in the pole climbing test (*p* < 0.0001, Fig. 2A, B). However, compared with MPTP and sham rTMS mice, MPTP + rTMS (10 Hz)-treated mice spent less time and displayed considerably improved performance on the pole test (34.97% decrease in rTMS-treated PD mice vs. sham-treated PD mice, *p* < 0.0001, Fig. 2A, B). However, the Treg inhibitor anti-CD25 significantly abolished this improvement (Fig. 2A, B).

### rTMS rescued dopaminergic neurons, reversed the decrease in the circulating proportion of Tregs and attenuated inflammatory cytokines in MPTP-induced mice

We performed immunohistochemical staining to examine the loss of dopaminergic neurons, BDNF and GDNF in the SN after MPTP administration. The immunohistochemical staining results showed that MPTP-induced mice showed 50.15%, 38.38% and 21.82% reductions in TH-positive neurons and fibers, BDNF and GDNF in the SN compared with saline normal control (NC) mice, respectively (*p* < 0.001 for TH, *p* < 0.001 for BDNF and GDNF; Supplementary Fig. 3). Compared with those in the MPTP and sham rTMS groups, TH, BDNF and GDNF were considerably increased in the MPTP + rTMS (10 Hz) group (Supplementary Fig. 3). Compared with that in saline NC mice, numerous microglia were activated in the SN of MPTP mice, and the proinflammatory cytokines IL-6, IFN-γ, TNF-α and IL-1β in the ventral midbrain of MPTP mice were significantly increased by 75.37%, 185.87%, 91.66%, 140.36%, respectively, while the anti-inflammatory cytokines IL-10 and TGF-β1 were significantly reduced by 59.90% and 17.02%, respectively (Fig. 2E-G). The results showed a significant decline in the circulating proportion of Treg cells (% CD4 + T cell population) in PD mice (1.98 ± 0.33%) compared to NC mice (4.54 ± 0.37%) (Fig. 2C, D). Compared with that in sham mice, rTMS (10 Hz) therapy significantly reversed the decrease in the circulating proportion of Tregs in PD mice (302.44% increase in rTMS-treated PD mice vs. sham-treated PD mice, *p* < 0.0001 for Tregs, Fig. 2C, D), increased the anti-inflammatory mediators IL-10 and TGF-β1 by 69.44% and 12.34%, respectively, deactivated microglia and decreased the inflammatory cytokines IL-6, IFN-γ, TNF-α and IL-1β by 28.41%, 24.40%, 30.48% and 22.78% in the ventral midbrain of PD mice, respectively (Fig. 2E-G). This finding indicated that rTMS treatment of PD mice could attenuate inflammation in the brain. Injection of the Treg inhibitor anti-CD25 (1 mg/kg/d, for 3 days) into PD mice significantly abolished the rTMS-mediated increase in circulating Tregs and anti-inflammatory cytokines and reversed the downregulation of these inflammatory cytokines, as well as microglial activation in the SN (*p* < 0.0001 for Tregs, *p* < 0.0001 for microglia, *p* < 0.0001 for inflammatory and anti-inflammatory cytokines; Fig. 2C-G).

### Quantitative proteomics analysis of Treg- and rTMS-related proteins

**Fig. 3:**
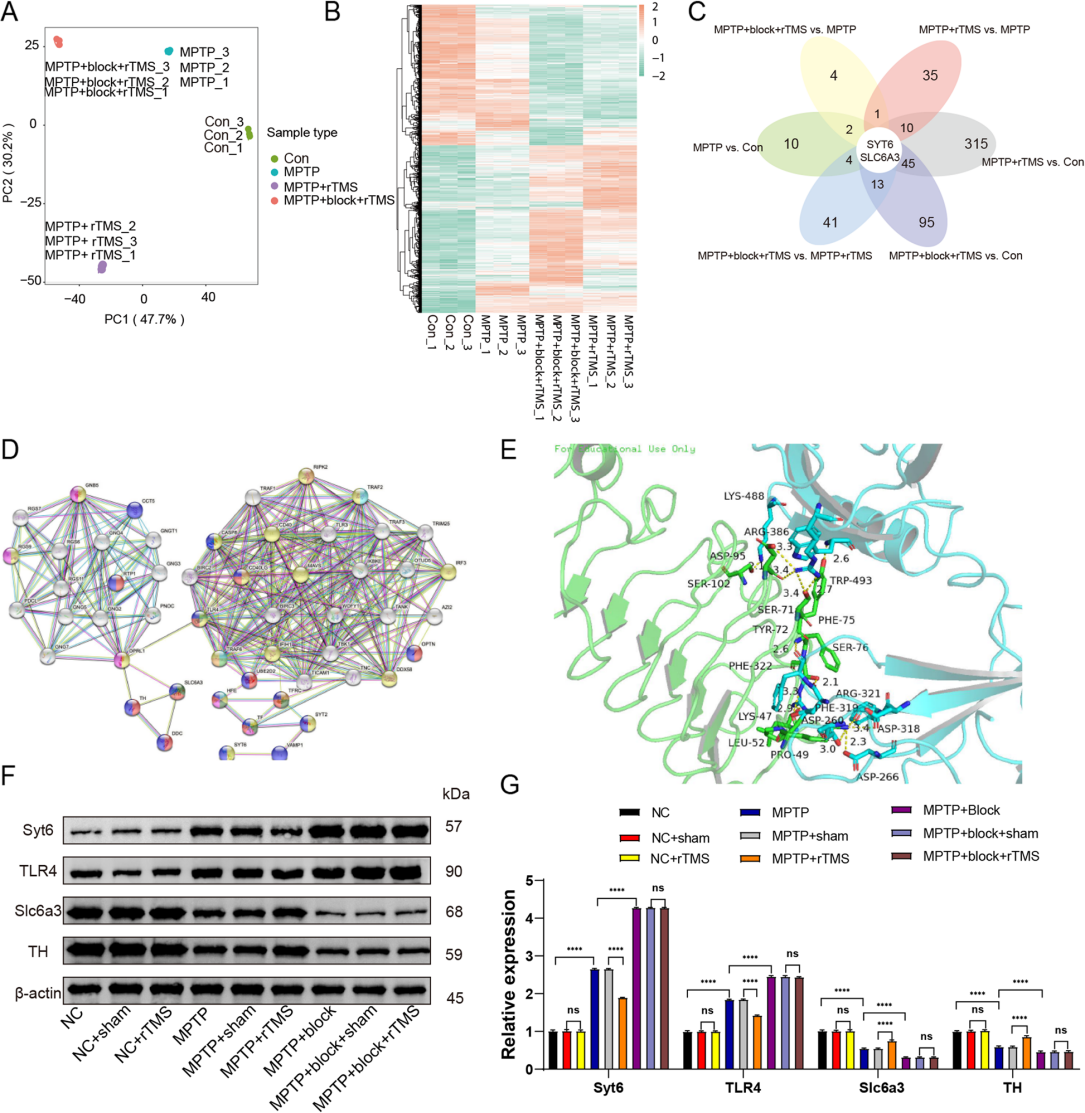
Quantitative proteomic analysis identified the Treg-related downstream targets Syt6, TLR4, TH and Slc6a3 that were associated with rTMS stimulation in PD. **A** Principal component analysis (PCA). The results showed an increased degree of aggregation among replicate samples and improved quantitative repeatability, with a large significant difference between the groups (*n* = 3, Con = saline normal control group, MPTP = MPTP-induced PD group, MPTP + rTMS = MPTP + rTMS treatment group, MPTP + block + rTMS = MPTP + Treg block + rTMS treatment group). **B** Heatmap showing the gene expression level per sample relative to the average expression of all samples. Red represents higher expression, and green represents lower expression (*n* = 3, Con = saline normal control group, MPTP = MPTP-induced PD group, MPTP + rTMS = MPTP + rTMS treatment group, MPTP + block + rTMS = MPTP + Treg block + rTMS treatment group). **C** All differentially expressed genes in each group were analyzed by intersection analysis, and two common differentially expressed genes, Slc6a3 and Syt6, were screened out (*n* = 3, Con = saline normal control group, MPTP = MPTP-induced PD group, MPTP + rTMS = MPTP + rTMS treatment group, MPTP + block + rTMS = MPTP + Treg block + rTMS treatment group). **D** Protein–protein interaction (PPI) network of differentially expressed proteins in the MPTP + rTMS and MPTP + block + rTMS groups (PPI enrichment p value < 1.0e-16); (*n* = 3, MPTP + rTMS = MPTP + rTMS treatment group, MPTP + block + rTMS = MPTP + Treg block + rTMS treatment group). **E** The SYT6 protein has a binding and interaction relationship with TLR4. **F** Representative western blot bands of the protein expression of Syt6, TLR4, TH, and Slc6a3 in the ventral midbrain of each group of mice. **G** Mean protein expression of Syt6, TLR4, TH, and Slc6a3 in the ventral midbrain in each group of mice ± SD. (Syt6: F8, 18 = 7696, NC vs. NC + sham: *P* > 0.9999; NC vs. MPTP: *P* < 0.0001; NC + sham vs. NC + rTMS: *P* > 0.9999; MPTP vs. MPTP + sham: *P* > 0.9999; MPTP vs. MPTP + rTMS: *P* < 0.0001; MPTP + sham vs. MPTP + rTMS: *P* < 0.0001; MPTP vs. MPTP + bock: *P* < 0.0001; MPTP + block + sham vs. MPTP + block + rTMS: *P* > 0.9999; TLR4: F8, 18 = 3511, NC vs. NC + sham: *P* > 0.9999; NC vs. MPTP: *P* < 0.0001; NC + sham vs. NC + rTMS: *P* > 0.9999; MPTP vs. MPTP + sham: *P* > 0.9999; MPTP vs. MPTP + rTMS: *P* < 0.0001; MPTP + sham vs. MPTP + rTMS: *P* < 0.0001; MPTP vs. MPTP + block: *P* < 0.0001; MPTP + block + sham vs. MPTP + block + rTMS: *P* = 0.9628; Slc6a3: F8, 18 = 626.5, NC vs. NC + sham: *P* > 0.9999; NC vs. MPTP: *P* < 0.0001; NC + sham vs. NC + rTMS: *P* > 0.9999; MPTP vs. MPTP + sham: *P* > 0.9999; MPTP vs. MPTP + rTMS: *P* < 0.0001; MPTP + sham vs. MPTP + rTMS: *P* < 0.0001; MPTP vs. MPTP + block: *P* < 0.0001; MPTP + block + sham vs. MPTP + block + rTMS: P > 0.9999; TH: F8, 18 = 334.50, NC vs. NC + sham: *P* = 0.9999; NC vs. MPTP: *P* < 0.0001; NC + sham vs. NC + rTMS: *P* = 0.9998; MPTP vs. MPTP + sham: *P* > 0.9999; MPTP vs. MPTP + rTMS: *P* < 0.0001; MPTP + sham vs. MPTP + rTMS: *P* < 0.0001; MPTP vs. MPTP + block: *P* < 0.0001; MPTP + block + sham vs. MPTP + block + rTMS: *P* > 0.9999, *n* = 3 mice per group). Data are presented as mean ± SD; One-way ANOVA with Fisher’s LSD multiple comparison post hoc test (**G**) ****P < 0.0001. Each data point represents an individual mouse. Comparisons with no asterisk had a *P* > 0.05 and were considered not significant

To further identify the molecular targets and hub proteins related to Tregs and rTMS therapy in PD, we used tandem mass tag (TMT) technology to label 12 samples (4 groups: saline normal control, MPTP, MPTP + rTMS, and MPTP + block + rTMS, 3 samples per group) for quantitative proteomics analysis. We first identified 7, 817 proteins (Supplementary Table 1), and proteins that were significantly upregulated and downregulated in all four clustered groups are shown in Supplementary Table 2A and Fig. 3A, B. We next identified 2 proteins (Syt6 and Slc6a3) through overlapping pairwise comparisons (Fig. 3C). Analysis of the protein–protein interaction (PPI) network also confirmed that Syt6 and Slc6a3 were involved in multiple pathways and associated with the activation of T cells (Fig. 3D). We performed full rigid-body docking orientation analysis of the two proteins with the ZDOCK algorithm and found that Syt6 docked with TLR4. This proteomics analysis suggests that there may be protein interactions between TLR4 and SYT6, and the two protein interactions may exert biological effects through multiple binding sites. The main sites of TLR4 are LYS-47, SEP-71 and ASP-95, and the main sites of SYT6 are ARG -321 and ARG-386 (Supplementary Table 2B, C). These proteins are considered hub proteins and may serve as novel targets of PD.

To further characterize the role of rTMS and identify its association with Tregs in PD, we investigated these screened hub proteins by Western blotting (Fig. 3F, G). Compared with those in saline normal controls, the levels of Syt6 and TLR4 were significantly increased by 164.19% and 85.55%, respectively, and TH and Slc6a3 were significantly decreased by 41.08% and 45.97% in the PD mouse brain, respectively, while rTMS therapy reversed these changes (*P* < 0.0001 for Syt6, TLR4, TH and Slc6a3; Fig. 3A-E). More interestingly, after inhibiting Tregs with anti-CD25 and stimulating PD mice with rTMS, the levels of Syt6, TLR4, TH and Slc6a3 in PD mouse brains were significantly reversed compared with those in the PD + rTMS group (*p* < 0.0001; Fig. 3F, G).

### Verification of the role of Treg and rTMS-related protein syt6 in MPTP mice by virus interference method

**Fig. 4:**
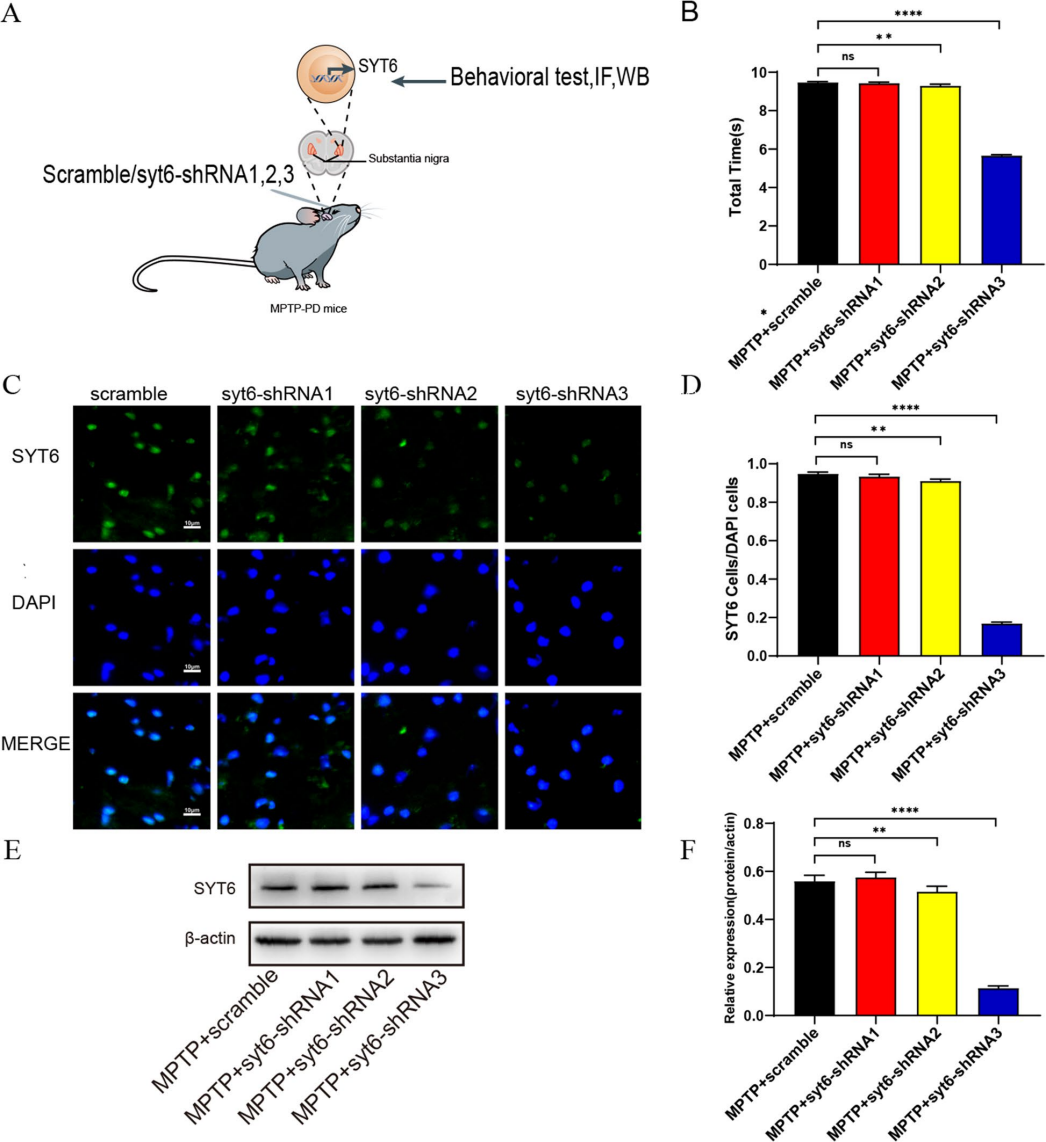
Screening out effective virus interference bands in syt6 to ensure interference efficiency. **A** Adeno-associated virus (AAV) vector was injected into midbrain via a stereotactic midbrain approach to block syt6 level. Pole climbing pole test, immunofluorescent staining and WB were used to detect the efficiency of transfection. **B** Quantification of total the times for each group mice that spent in pole climbing test. (F3,8 = 3691, MPTP + scramble vs. MPTP + syt6-shRNA1: *P* = 0.8070; MPTP + scramble vs. MPTP + syt6-shRNA2:*P* = 0.0022; MPTP + scramble vs. MPTP + syt6- shRNA3: *P* < 0.0001; *n* = 3 mice per group). **C** Immunofluorescent staining images of Syt6 expression in the SN of each group mice. Scale bar, 10 μm. **D** Quantitative analyses of Syt6 immunostaining in the SN. (F3,8 = 4701, MPTP + scramble vs. MPTP + syt6-shRNA1: *P* = 0.3061; MPTP + scramble vs. MPTP + syt6-shRNA2: *P* = 0.0040; MPTP + scramble vs. MPTP + syt6-shRNA3: *P* < 0.0001; *n* = 3 mice per group). **E** Representative western blot bands of the protein expression of Syt6 in the ventral midbrain of each group mice. **F** Quantitative analysis of the protein expression of Syt6 in the ventral midbrain of each group mice. (F3,8 = 275.9, MPTP + scramble vs. MPTP + syt6-shRNA1: *P* = 0.8026; MPTP + scramble vs. MPTP + syt6-shRNA2: *P* = 0.0018; MPTP + scramble vs. MPTP + syt6- shRNA3: *P* < 0.0001; *n* = 3 mice per group). Data are presented as mean ± SD; One-way ANOVA with Fisher’s LSD multiple comparison post hoc test (**G**). ****P < 0.0001, *P < 0.05. Each data point represents an individual mouse. Comparisons with no asterisk had a *P* > 0.05 and were considered not significant

**Fig. 5:**
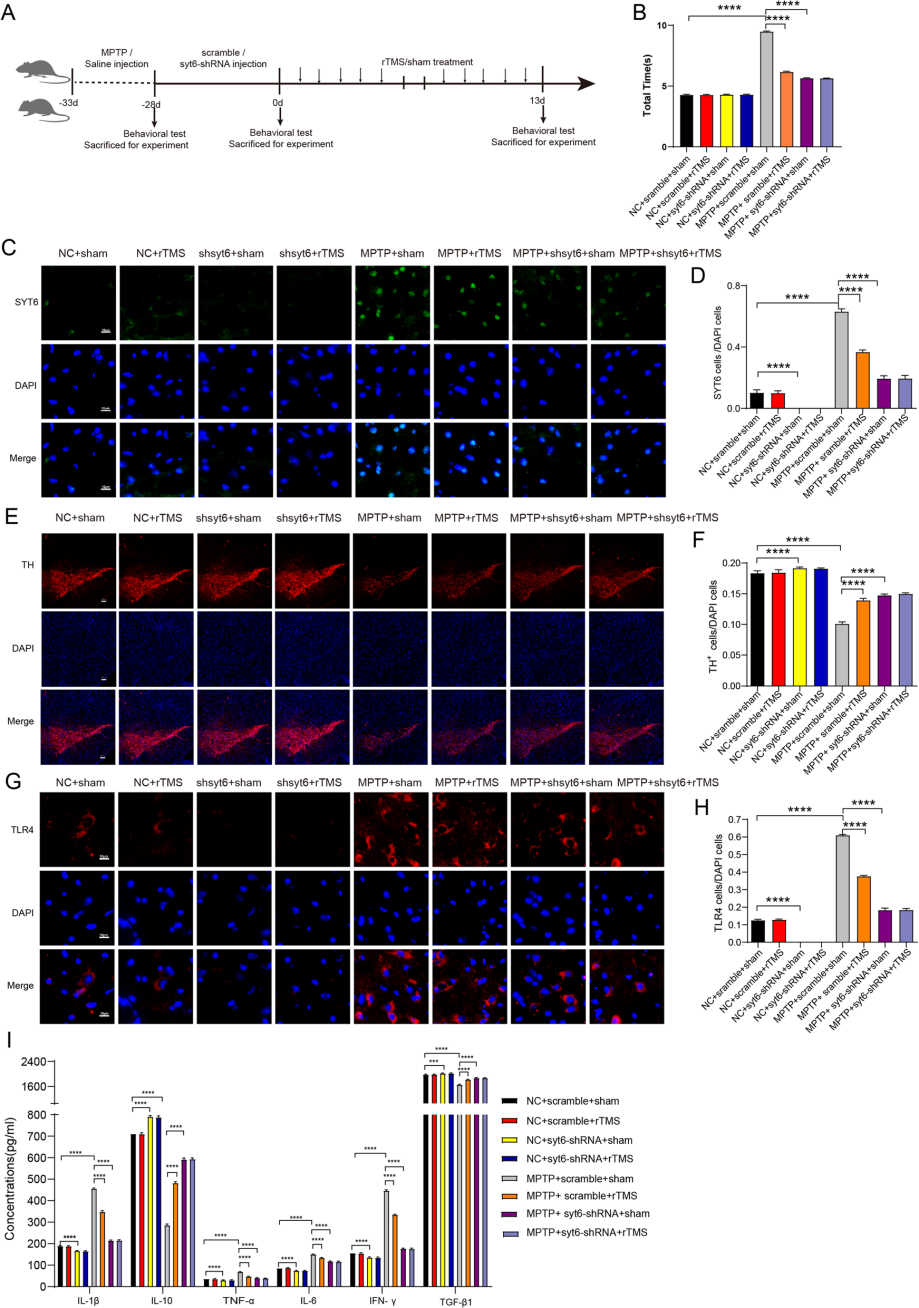
Verification of the role of Treg and rTMS-related protein syt6 in MPTP mice by virus interference syt6 method. **A** Animal experimental design and virus interference syt6 scheme to explore the role of Treg and rTMS-related protein syt6 in the MPTP-induced PD mouse model. **B** Quantification of total the times for each group mice that spent in pole climbing test. (F7,112 = 18,690, NC + scramble + sham vs. NC + scramble + rTMS: *P* > 0.9999; NC + sramble + sham vs. MPTP + scramble + sham: *P* < 0.0001; NC + syt6-shRNA + sham vs. NC + syt6-shRNA + rTMS: *P* = 0.9999; MPTP + scramble + sham vs. MPTP + scramble + rTMS: *P* < 0.0001; MPTP + scramble + sham vs. MPTP + syt6-shRNA + sham: *P* < 0.0001; MPTP + syt6-shRNA + sham vs. MPTP + syt6-shRNA + rTMS: *P* = 0.9999, *n* = 15 mice per group). **C** Representative images of SYT6 expression in the SN. Scale bar, 10 μm. **D** Quantitative analyses of SYT6 immunostaining in the SN. (F7,72 = 2866, NC + scramble + sham vs. NC + scramble + rTMS: *P* > 0.9999; NC + scramble + sham vs. NC + syt6-shRNA + sham: *P* < 0.0001; NC + scramble + sham vs. MPTP + scramble + sham: *P* < 0.0001; NC + syt6-shRNA + sham vs. NC + syt6-shRNA + rTMS: *P* > 0.9999; MPTP + scramble + sham vs. MPTP + scramble + rTMS: *P* < 0.0001; MPTP + scramble + sham vs. MPTP + syt6-shRNA + sham: *P* < 0.0001; MPTP + syt6-shRNA + sham vs. MPTP + syt6- shRNA + rTMS: *P* > 0.9999, *n* = 10 mice per group). **E** Representative images of TH expression in the SN. Scale bar, 50 μm. **F** Quantitative analyses of TH immunostaining in the SN. (F7,72 = 649.7, NC + sramble + sham vs. NC + scramble + rTMS: *P* = 0.9426; NC + sramble + sham vs. NC + syt6-shRNA + sham: *P* < 0.0001; NC + scramble + sham vs. MPTP + scramble + shamm: *P* < 0.0001; NC + syt6-shRNA + sham vs. NC + syt6-shRNA + rTMS: *P* = 0.9993; MPTP + scramble + sham vs. MPTP + scramble + rTMS: *P* < 0.0001; MPTP + scramble + sham vs. MPTP + syt6-shRNA + sham: *P* < 0.0001; MPTP + syt6-shRNA + sham vs. MPTP + syt6- shRNA + rTMS: *P* > 0.9999, *n* = 10 mice per group). **G** Representative images of TLR4 expression in the SN. Scale bar, 10 μm. **H** Quantitative analyses of TLR4 immunostaining in the SN. (F7,72 = 1923, NC + sramble + sham vs. NC + scramble + rTMS: *P* > 0.9999; NC + scramble + sham vs. NC + syt6-shRNA + sham: *P* < 0.0001; NC + scramble + sham vs. MPTP + scramble + sha-m: *P* < 0.0001; NC + syt6-shRNA + sham vs. NC + syt6-shRNA + rTMS: *P* > 0.9999; MPTP + scramble + sham vs. MPTP + sramble + rTMS: *P* < 0.0001; MPTP + scramble + sham vs. MPTP + syt6-shRNA + sham: *P* < 0.0001; MPTP + syt6-shRNA + sham vs. MPTP + syt6- shRNA + rTMS: *P* > 0.9999, *n* = 10 mice per group). **I** ELISA was used to analyze the protein expression of IL-1β, TNF-α, IL-6, IL-10, TGF-β1 and IFN-γ in the ventral midbrain in each group of mice. (IL-1β: F7,40 = 6230, NC + scramble + sham vs. NC + scramble + rTMS: *P* = 0.9958; NC + scramble + sham vs. NC + syt6-shRNA + sham: *P* < 0.0001; NC + scramble + sham vs. MPTP + scramble + sham: *P* < 0.0001; NC + syt6-shRNA + sham vs. NC + syt6-shRNA + rTMS: *P* = 0.9279; MPTP + scramble + sham vs. MPTP + scramble + rTMS: *P* < 0.0001; MPTP + scramble + sham vs. MPTP + syt6-shRNA + sham: *P* < 0.0001; MPTP + syt6-shRNA + sham vs. MPTP + syt6-shRNA + rTMS: *P* = 0.9998; TNF-α: F7,40 = 347.7, NC + scramble + sham vs. NC + scramble + rTMS: *P* = 0.9651; NC + scramble + sham vs. NC + syt6-shRNA + sham: *P* < 0.0001; NC + scramble + sham vs. MPTP + scramble + sham: *P* < 0.0001; NC + syt6-shRNA + sham vs. NC + syt6-shRNA + rTMS: *P* > 0.9999; MPTP + scramble + sham vs. MPTP + scramble + rTMS: P < 0.0001; MPTP + scramble + sham vs. MPTP + syt6-shRNA + sham: *P* < 0.0001; MPTP + syt6-shRNA + sham vs. MPTP + syt6-shRNA + rTMS: *P* = 0.8441; IL-6: F7,40 = 1523, NC + scramble + sham vs. NC + scramble + rTMS: *P* = 0.8101; NC + scramble + sham vs. NC + syt6-shRNA + sham: *P* < 0.0001; NC + scramble + sham vs. MPTP + scramble + sham: *P* < 0.0001; NC + syt6-shRNA + sham vs. NC + syt6-shRNA + rTMS: *P* = 0.9999; MPTP + scramble + sham vs. MPTP + scramble + rTMS: *P* < 0.0001; MPTP + scramble + sham vs. MPTP + syt6-shRNA + sham: *P* < 0.0001; MPTP + syt6-RNA + sham vs. MPTP + syt6-shRNA + rTMS: *P* = 0.4726; IL-10: F7,40 = 4702, NC + scramble + sham vs. NC + scramble + rTMS:*P* > 0.9999; NC + scramble + sham vs. NC + syt6-shRNA + sham: *P* < 0.0001; NC + scramble + sham vs. MPTP + scramble + sham: *P* < 0.0001; NC + syt6-shRNA + sham vs. NC + syt6-shRNA + rTMS: *P* = 0.9894; MPTP + scramble + sham vs. MPTP + scramble + rTMS: *P* < 0.0001; MPTP + scramble + sham vs. MPTP + syt6-shRNA + sham: *P* < 0.0001; MPTP + syt6-shRNA + sham vs. MPTP + syt6-shRNA + rTMS: *P* = 0.9997; TGF-β1: F7,40 = 482.6, NC + scramble + sham vs. NC + scramble + rTMS: *P* > 0.9999; NC + scramble + sham vs. NC + syt6-shRNA + sham: *P* = 0.0004; NC + scramble + sham vs. MPTP + scramble + sham: *P* < 0.0001; NC + syt6-shRNA + sham vs. NC + syt6-shRNA + rTMS:*P* > 0.9999; MPTP + scramble + sham vs. MPTP + scramble + rTMS: *P* < 0.0001; MPTP + scramble + sham vs. MPTP + syt6-shRNA + sham: *P* < 0.0001; MPTP + syt6-shRNA + sham vs. MPTP + syt6-shRNA + rTMS: *P* > 0.9999; IFN-γ: F7,40 = 6016, NC + scramble + sham vs. NC + scramble + rTMS: *P* = 0.9784; NC + scramble + sham vs. NC + syt6-shRNA + sham: *P* < 0.0001; NC + scramble + sham vs. MPTP + scramble + sham: *P* < 0.0001; NC + syt6-shRNA + sham vs. NC + syt6-shRNA + rTMS: P > 0.9999; MPTP + scramble + sham vs. MPTP + scramble + rTMS: *P* < 0.0001; MPTP + scramble + sham vs. MPTP + syt6-shRNA + sham: *P* < 0.0001; MPTP + syt6-shR-NA + sham vs. MPTP + syt6-shRNA + rTMS: *P* = 0.9890; *n* = 6 mice per group). Data are presented as mean ± SD; One-way ANOVA with Fisher’s LSD multiple comparison post hoc test (**B**, **D**, **F**, **H**, **I**). ****P < 0.0001. Each data point represents an individual mouse. Comparisons with no asterisk had a *P* > 0.05 and were considered not significant

Adeno-associated virus (AAV) vector was injected into midbrain via a stereotactic midbrain approach to block syt6 level. Immunofluorescent staining and WB were used to detect the efficiency of transfection (Fig. 4A, C, E). Compared with those in MPTP + scramble control group, the SYT6/DAPI expression was significantly decreased by 82.2% in the SN of MPTP + syt6-shRNA3 group, and the syt6 relative expression level was similar to that in WB. Similarly, mice in the MPTP + Syt6-shRNA3 group showed significantly improved performance in pole test after Syt6 knockdown in SN (Fig. 4B, D, F). Therefore, we then used syt6-shRNA3 as an AAV to block the expression of syt6 protein in SN. Interestingly, rTMS did not change the total climbing time of MPTP mice with syt6 AAV. On the contrary, rTMS treatment significantly reduced the total climbing time of MPTP mice with AAV empty vector (Fig. 5A, B). We next examined the expression of syt6, TLR4, TH and inflammatory cytokines with syt6 AAV intervention in MPTP-induced mice with or without rTMS treatment (Fig. 5C, E, G, I). There was a significant increase by 47.72% in TH expression in MPTP mice with syt6 AAV intervention but rTMS treatment did not change the TH level in MPTP mice with syt6 AVV intervention. On the contrary, rTMS treatment significantly increased TH level in MPTP mice with AAV empty vector by 39.51% (Fig. 5E, F). There was a significant decrease by 80.47% of syt6 expression in MPTP mice with syt6 AAV intervention (Fig. 5C, D); however, rTMS did not change the syt6, TLR4, IL-β1, TNF-α and IL-6 levels in MPTP mice with syt6 AAV. On the contrary, rTMS significantly decreased TLR4, IL-β1, TNF-α and IL-6 level by 41.80%, 25.52%, 32.12% and 10.79% respectively in MPTP mice with AAV empty vector (Fig. 5G, H, I). In addition, the levels of anti-inflammatory cytokines, such as IL-10 and TGF-β, were significantly elevated in MPTP-induced mice following rTMS treatment, which was not altered by Syt6 knockdown (Fig. 5I), Furthermore, Syt6 expression was detected in Iba1-positive cells (Supplementary Fig. 4). These data suggest that rTMS treatment affects syt6, a hub protein, to increase the proportion of Tregs in MPTP mice.

## Discussion

Our randomized sham controlled clinical trial demonstrated that 10 days of rTMS stimulation improved motor dysfunction in PD subjects after 13 days, and the effect lasted for 40 days**.** Importantly, rTMS treatment elevated peripheral Treg levels in PD patients (*p* < 0.0001), but not in sham group (Fig. 1G). In addition, we found a significant negative correlation between the increased proportion of peripheral blood Tregs and MDS-UPDRS III scores (Fig. 1J), suggesting that motor improvement induced by rTMS might be due to an increase in circulating Tregs.

rTMS is a non-invasive neuromodulation technique that utilizes magnetic fields to stimulate nerve cells in the brain. Extensive research has been conducted on its potential therapeutic effects in various neurological and psychiatric disorders, including Alzheimer's disease, PD, stroke, and multiple sclerosis. Preclinical studies have suggested that rTMS may improve cognitive function in Alzheimer's disease by enhancing synaptic plasticity and reducing amyloid-beta plaques. rTMS has also been studied for its potential to improve motor function and reduce fatigue in multiple sclerosis patients by modulating the excitability of the motor cortex [46]. A systematic review has shown that rTMS intervention may have the potential to modify apathy among patients with chronic stroke [46]. The exact mechanisms by which rTMS exerts its effects are not fully understood but are normally thought to involve changes in neuronal activity, synaptic plasticity, and neurotransmitter release. In our study, we found that rTMS improves motor function by modulating the function of Tregs and suppressing toxic neuroinflammation in PD, which is consistent with previous studies [47–49]. Ongoing research is exploring the use of rTMS in combination with other neuromodulation techniques to enhance therapeutic effects, indicating the potential therapeutic effects of rTMS in the treatment of neurodegenerative diseases.

Evidence-based guidelines for the therapeutic use of rTMS have demonstrated that high-frequency rTMS (HF-rTMS) applied to bilateral M1 regions or the left dorsolateral prefrontal cortex (DLPFC) can ameliorate motor impairment and depression, respectively, in Parkinson's disease, with Level B evidence indicating probable efficacy [24]. Furthermore, a randomized controlled trial published in a neurology journal provides Class I evidence that, in patients with Parkinson's disease experiencing depression, bilateral M1 rTMS (comprising 1,000 stimuli for each M1; 50 trains of 4 s at 10 Hz with 40 stimuli per train) results in enhanced motor function compared to sham rTMS [50]. Additionally, a meta-analysis has shown that multiple sessions of HF-rTMS over the M1, particularly when administered bilaterally, with a cumulative total of 18,000–20,000 pulses, appears to be the optimal parameters for improving motor symptoms in Parkinson's disease [25]. Informed by these references, our clinical trial was designed to ameliorate the motor symptoms of Parkinson's disease using HF-rTMS applied to both M1 areas, delivering 1,000 stimuli to each side. The treatment spanned 10 days, accumulating a total of 20,000 stimuli.

To take our findings further, we next attempted to validate our clinical findings in a PD animal model. Previous studies have indicated that induction of Tregs promoted neuronal survival through suppression of microglial activation in MPTP-induced PD mouse model [19, 51]. Here, we utilized MPTP PD mice (Fig. 2A, B; Supplementary Fig. 1A, B) and found that Tregs in the spleen were dysregulated in these mice compared with NC mice (Fig. 2C, D), which is consistent with previous reports [19, 52]. Compared with that in NC mice, numerous microglia in the SN of PD mice were activated, and the proinflammatory cytokines IL-6, IFN-γ, TNF-α and IL-1β were significantly increased, while the anti-inflammatory cytokines IL-10 and TGF-β1 were significantly reduced (Fig. 2E-G). rTMS has been shown to ameliorate inflammation and regulate neurotrophic factors in depression, anxiety, cerebral ischemia and spinal cord lesions [53–56]. To evaluate whether rTMS could influence PD by impacting circulating Tregs and regulating inflammation in the brain, we applied rTMS to the brains of MPTP mice for 10 days and identified that a 10 Hz stimulation led to consistent and immediate improvements in motor symptoms at 1 day after rTMS therapy (Fig. 2A, B). In comparison to the sham-treated mice, our findings revealed that rTMS at 10 Hz significantly enhanced the circulating proportion of Tregs and TH + neurons within the SN (Fig. 2C, D, Supplementary Fig. 1A, B), increased the anti-inflammatory mediators IL-10 and TGF-β1, deactivated microglia and decreased the inflammatory cytokines IL-6, IFN-γ, TNF-α and IL-1β1 in the ventral midbrain of PD mice (*p* < 0.0001, Fig. 2E-G). These results suggest that rTMS therapy could attenuate inflammation in the brains in PD mice. One previous study showed that the transfer of Tregs to MPTP mice prevented DA neuronal degeneration by suppressing microglial activation and attenuating neuroinflammation, suggesting that the increased Tregs exerted neuroprotective effects on PD [57]. We next injected the Treg inhibitor anti-CD25 into MPTP mice and observed that this treatment significantly attenuated the rTMS-mediated improvements in motor symptoms, abolished the rTMS-mediated upregulation of circulating Tregs, and reversed the downregulation of inflammatory cytokines in the ventral midbrain and microglial activation in the SN (Fig. 2C-G). These results indicate that the restoration of peripheral Tregs may be pivotal following rTMS stimulation in the brain, and rTMS may contribute to neuroprotection in PD at least partially by upregulating peripheral Tregs, subsequently reducing neuroinflammation and deactivating microglia in the SN. rTMS also profoundly upregulated the levels of TH, BDNF and GDNF in the SN (Supplementary Fig. 3), and these effects were partially abolished by anti-CD25 (Supplementary Fig. 3).

To further identify the hub proteins related to Tregs and to screen biological indicators, tandem mass tags (TMT) technology was used to label 12 samples that were used for quantitative proteomic analysis. After intersection analysis of differentially expressed genes in the MPTP + block + rTMS treatment group and MPTP + rTMS group was performed, 30 differentially expressed genes were selected for pathway enrichment analysis, and a protein–protein interaction (PPI) network was constructed and identified the Treg-related downstream targets Syt6, TLR4, TH and Slc6a3 (Fig. 3A-E), which were closely associated with the activation of T cells after rTMS stimulation in MPTP mouse brain, Compared with those in the saline normal controls, the levels of Syt6 and TLR4 were significantly increased, and TH and Slc6a3 were significantly decreased in the MPTP mouse brain, while rTMS therapy reversed these effects (Fig. 3A-E). Interestingly, after inhibiting Tregs with anti-CD25 and administering rTMS stimulation, the changes in the levels of Syt6, TLR4, TH and Slc6a3 in MPTP mouse brains were significantly reversed compared with those in the PD + rTMS group (Fig. 3F,G). These results suggest that Syt6, TLR4, and Slc6a3 are key targets of Tregs in regulating MPTP following rTMS stimulation, further indicating an important pathophysiologic role of Tregs in rTMS treatment in PD. We also performed a full rigid-body docking orientation between two proteins by ZDOCK algorithm, and found that Syt6 was docking with TLR4 (Fig. 3E). These four proteins were recognized as the hub node proteins and may serve as novel targets in regulating PD.

Four identified proteins in our study exhibit close intersection with neuroinflammation, microglia and Tregs in neurodegeneration. Microglia and Tregs are both key players in the immune response and homeostasis maintain within the CNS. Recent studies have highlighted that infiltrating Treg cells is essential for behavioral recovery and brain repair with their immunomodulatory effects on microglia after stoke [58]. A clinical study revealed a diminished proportion of Th2, Th17, and Treg cells in the peripheral blood of PD patients, along with a reduction in circulating CD4^+^T lymphocytes [29]. Collectively, Treg cells can modulate microglial responses, potentially leading to improved outcomes in neurodegenerative diseases. Here in our study, following rTMS treatment, we observed an increased proportion of aTreg/nTreg cells in the peripheral blood, accompanied by a decrease in pro-inflammatory factors such as IFN-γ, TNF-α, and IL-17α.

Tyrosine hydroxylase (TH), a tetrahydrobiopterin (BH4)-requiring monooxygenase that catalyzes the first and rate-limiting step in the biosynthesis of catecholamines (CAs), such as dopamine, has been suggested as the enzyme being a source of reactive oxygen species (ROS) in vitro and a target for radical-mediated oxidative injury [59]. A translational study highlighted that TLR4-mediated neuroinflammation plays an important role in intestinal and/or brain inflammation, which may be one of the key factors leading to neurodegeneration [60]. An animal study also revealed that TLR4 stimulates release of cytokine through NF-kB by activating glial cells, thus resulting in the death of dopaminergic neurons in the MTPT-induced mice [61]. One recent study showed that ex vivo expanded Tregs suppressed neuroinflammation, down-regulated the expression of TLR4 and alleviated AD pathology in vivo [62].

Syt6 (Synaptotagmin 6), one isoform of Synaptotagmins and a brain-specific Ca2 + /phospholipid-binding protein, has been shown to be a key component of the secretory machinery involved in acrosomal exocytosis and regulation of membrane trafficking in neurodegeneration [63, 64]. Its function is seldom investigated in neurological diseases; one recent report suggests that it is a key mediator of endocytic pathways in BDNF release [65]. SLC6A3, the gene encoding the dopamine transporter (DAT), is deeply influenced by neuroinflammation in Parkinson’s disease [30, 66]. Our results strongly suggest that rTMS exerts a beneficial effect on the peripheral immune system in PD via Tregs.

Next, to better understand the role of hub node proteins related to the therapeutic effect of rTMS in MPTP mice, we injected RNAi mediated AAV vector into the midbrain to block the hub protein in SN. Among Treg-related downstream targets Syt6, TLR4, TH and Slc6a3, previous studies have shown TLR4, TH and Slc6a3 have been associated with the pathogenesis of PD [6, 66–68]. Using the ZDOCK algorithm method, we found that Syt6 docked with TLR4, which has been reported to be associated with inflammation in SN of MPTP mice in previous studies [67–69]. We next selected virus interference with syt6 to verify if syt6 is the hub node protein related to the role of Treg in MPTP mice. We found that rTMS treatment did not change the total climbing time of MPTP mice, neither nigral IL-β1, TNF-α and IL-6 levels following syt6 virus interference, further verifying our hypothesis that syt6 participates in the therapeutic effect of rTMS in MPTP mice. To our knowledge, this is the first study directly showing SYT6 is involved in the neuro-pathogenesis of PD, especially modulating Treg-related neuro-inflammation.

Proinflammatory mediators and reactive oxygen species (ROS) in the circulation are closely associated with PD severity [5, 6, 70–72]. It is possible that the infiltration of circulating immune cells into the brain leads to extensive BBB breakdown, which in return further exacerbates peripheral inflammatory cytokines crossing the BBB and damaging neurons [13]. Among immune cells, Tregs play a vital role in regulating inflammatory responses and maintaining homeostasis in the CNS microenvironment [13]. One recent clinical trial with long-term sargramostim treatment for PD showed that the drug (a recombinant granulocyte macrophage colony-stimulatory factor) was able to stabilize immune homeostasis, restore peripheral immune function and increase the numbers of Tregs in the circulation [73]. Taken together, our findings (Fig. 1J) suggest that this restoration of peripheral blood Tregs in PD patients by rTMS stimulation may partially contribute to the improvement in motor dysfunction in PD patients.

## Limitations

Our data did not examine a prospective temporal relationship between motor improvement and the proportion of Tregs and hence longitudinal studies are needed to determine if the circulating Treg level following rTMS stimulation can be used to monitor the clinical progression in PD. Randomized trials of potent reversible pharmacological inhibitors or agonists of Tregs may help to clarify whether modifying Tregs can delay the progression of sporadic PD in patients.

## Conclusion

Our clinical and laboratory studies in PD patients and PD animal model suggest that rTMS improved motor function through modulating the function of Tregs and suppressing toxic neuroinflammation. We also identified several hub node proteins (especially Syt6) that may be potential therapeutic targets. Our findings provide pathophysiologic insights into the neuromodulatory effect of rTMS therapy in PD. Strategies to enhance peripheral Tregs and candidate molecular targets should be further explored.

## Supplementary Information

Supplementary Material 1.

Supplementary Material 2.

Supplementary Material 3.

Supplementary Material 4.

Supplementary Material 5.

Supplementary Material 6.

## Data Availability

The data that support the findings of this study are available from the corresponding author, upon reasonable request.

## Glossary

AAV: Adeno-associated virus
AD: Alzheimer’s disease
ALS: Amyotrophic lateral sclerosis
aTreg: Activated Treg
BBB: Blood-brain barrier
BDNF: Brain derived neurotrophic factor
CNS: Central Nervous System
DA: Dopaminergic
DAT: Dopamine transporter
ELISA: Enzyme-linked immunosorbent assay
PFA: 4% paraformaldehyde
GDNF: Glial cell line derived neurotrophic factor
H & Y: Hoehn-Yahr
IFN-γ: Interferon-γ
IL-1β: Interleukin-1β
IL-10: Interleukin-10
IL-6: Interleukin-6
LSD: Least significant difference
M1: primary motor cortex
MDS-UPDRS III: Movement Disorder Society Unified Parkinson's Disease Rating Scale part III
MFI: Median Fluorescence Intensity
MPTP: 1-methyl-4-phenyl-1, 2, 3, 6-tetrahydropyridine
NC: Normal control
nTreg: Naive Treg
PD: Parkinson disease
PDS: Parkinson’s Disease Syndrome
PDB: Protein data bank
PPI: Protein-protein interaction
rTMS: Repetitive transcranial magnetic stimulation
ROS: Reactive oxygen species
RMT: Resting motor threshold
RNAi: RNA interference
SNc: Substantia nigra pars compacta
Syt6: Synaptotagmin IV
SLC6A3: Solute carrier family 6 member 3
SD: Standard Deviation
Tregs: Regulatory T-cells
TNF-α: Tumor necrosis factor-alpha
TGF-β1: Transforming growth factor-β1
TH: Tyrosine hydroxylase
TMT: Tandem Mass Tags
TLR4: Toll-like Receptor 4
