# Supplementary figures and images for: Repetitive transcranial magnetic stimulation alleviates motor impairment in Parkinson’s disease: association with peripheral inflammatory regulatory T-cells and SYT6
Source: Mol Neurodegener. 2024 Oct 25;19:80. doi: 10.1186/s13024-024-00770-4 (PMC11515224; doi:10.1186/s13024-024-00770-4)

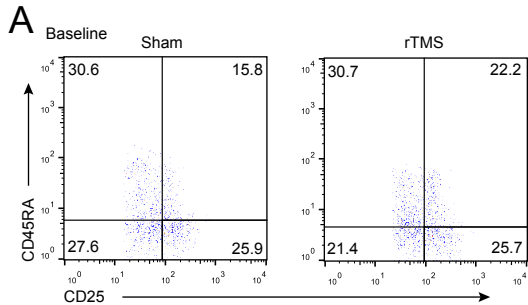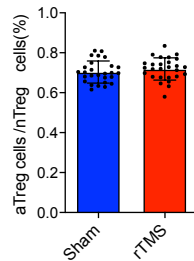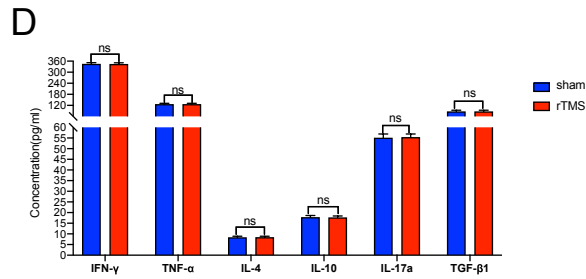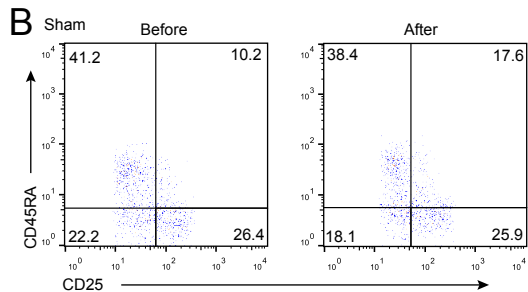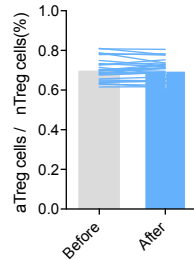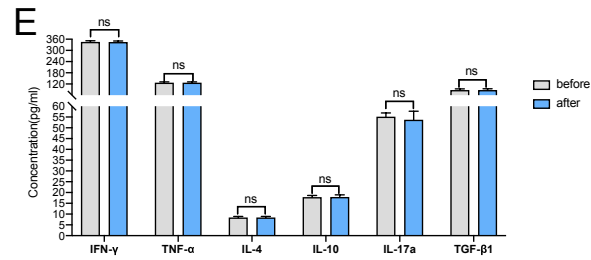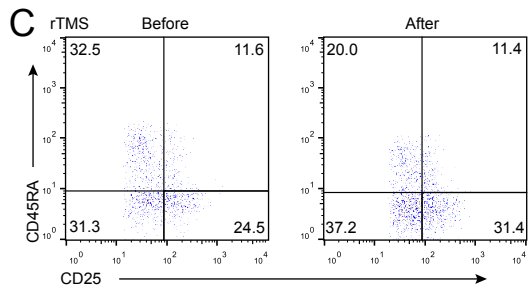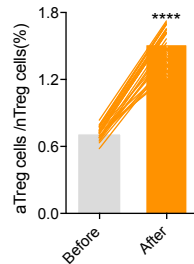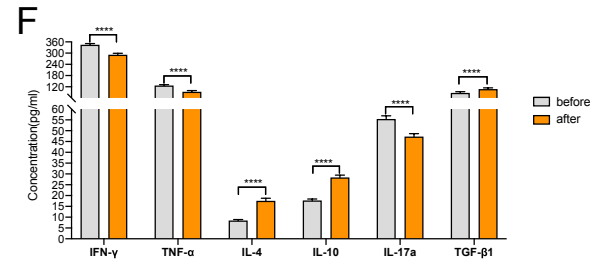

Supplement: Supplementary file 1 — Supplementary Material 1. [file 13024_2024_770_MOESM1_ESM.pdf]

A

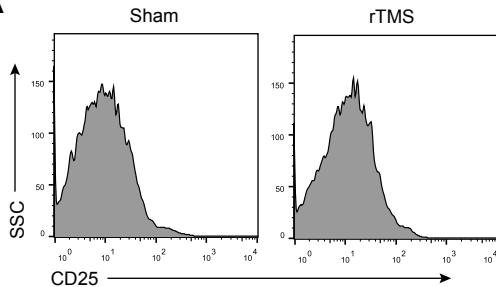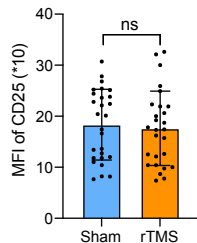

B

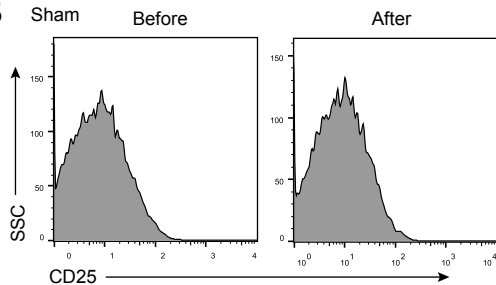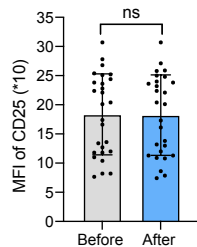

C

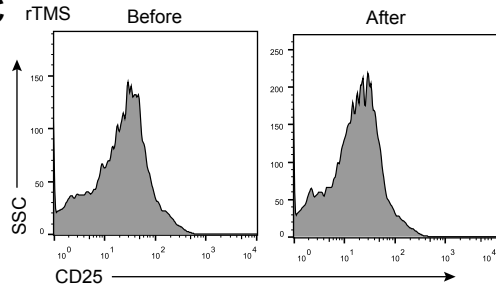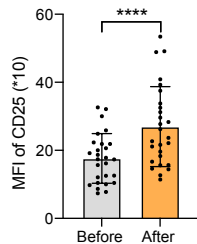

Supplement: Supplementary file 2 — Supplementary Material 2. [file 13024_2024_770_MOESM2_ESM.pdf]

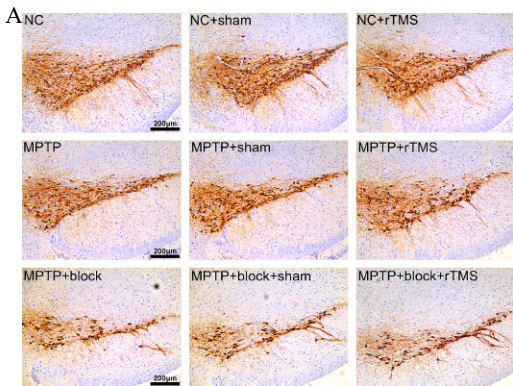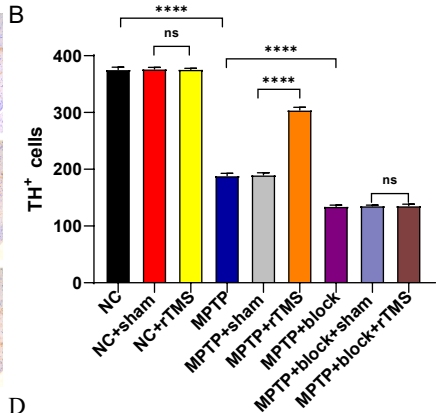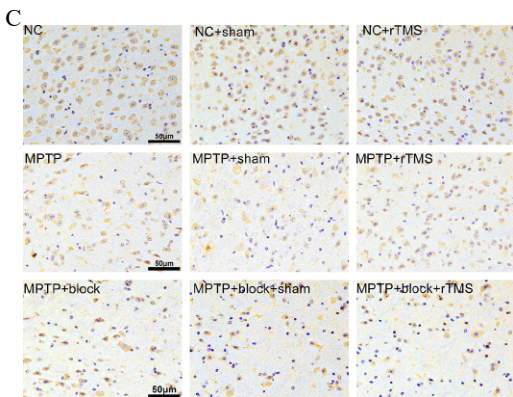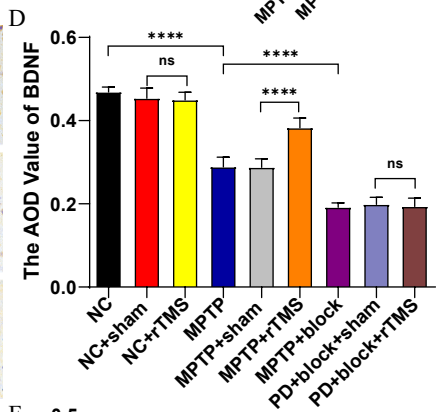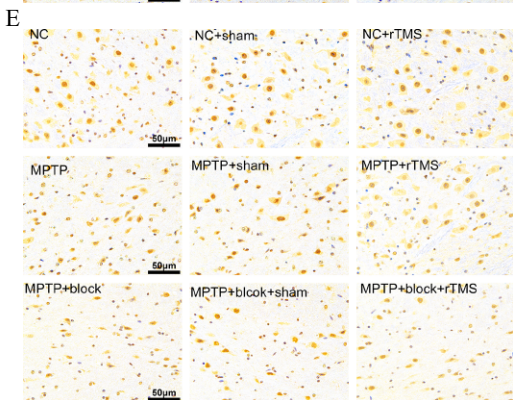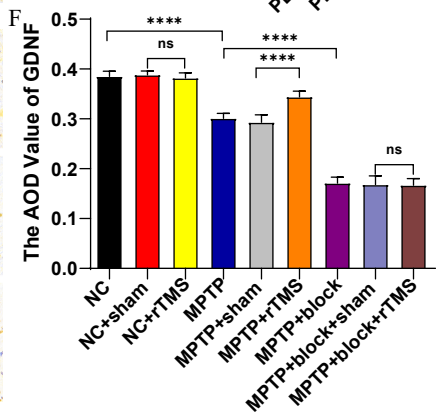

Supplement: Supplementary file 3 — Supplementary Material 3. [file 13024_2024_770_MOESM3_ESM.pdf]

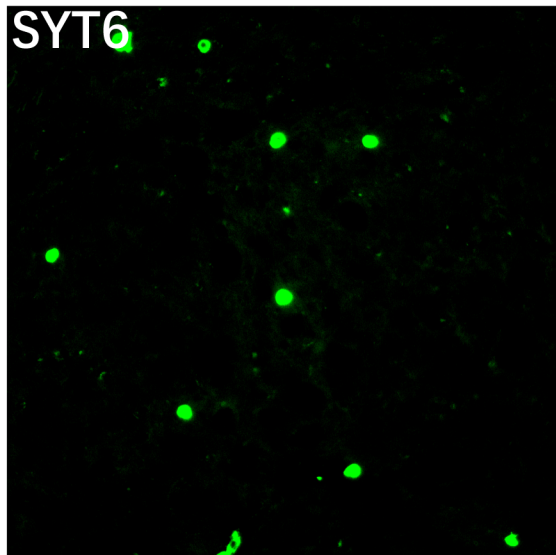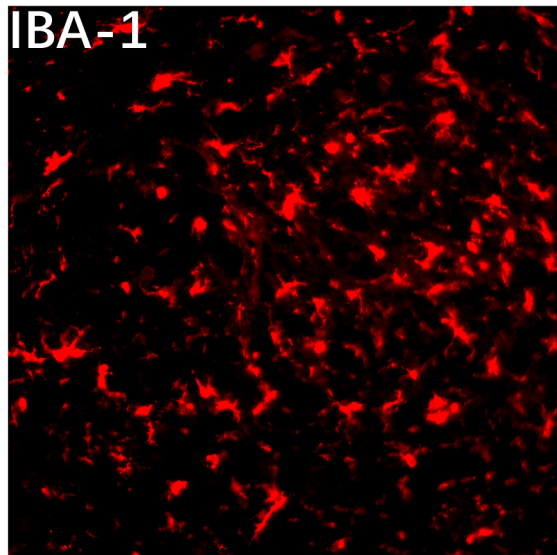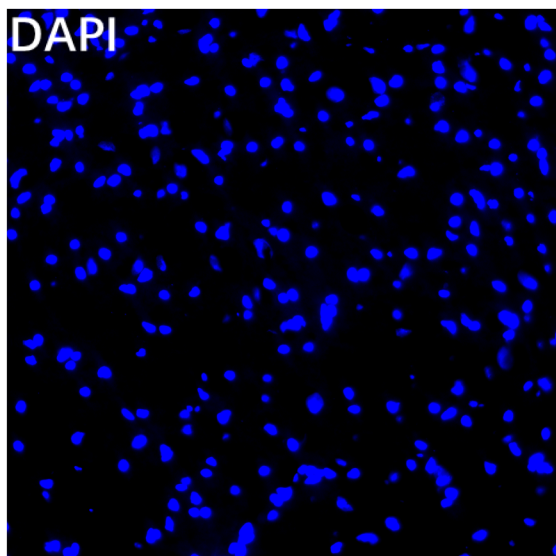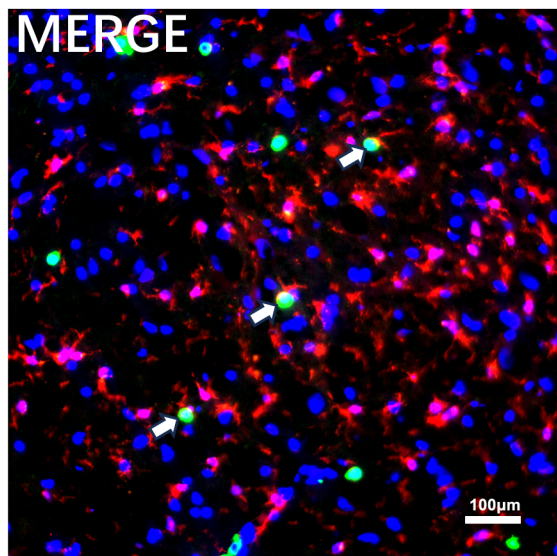

Supplement: Supplementary file 4 — Supplementary Material 4. [file 13024_2024_770_MOESM4_ESM.pdf]
