# Supplementary material for: Repetitive transcranial magnetic stimulation alleviates motor impairment in Parkinson’s disease: association with peripheral inflammatory regulatory T-cells and SYT6
Source: Mol Neurodegener. 2024 Oct 25;19:80. doi: 10.1186/s13024-024-00770-4 (PMC11515224; doi:10.1186/s13024-024-00770-4)
Supplement: Supplementary file 5 — Supplementary Material 5. [file 13024_2024_770_MOESM5_ESM.doc]

**Supplementary Table 1. Overview of protein identification**

| Title | Number |
| --- | --- |
| Total spectrums | 918595 |
| Matched spectrums | 279003 |
| Peptides | 157698 |
| Unique peptides | 150230 |
| Identified proteins | 7817 |
| Quantifiable proteins | 6956 |
