# Supplementary material for: Repetitive transcranial magnetic stimulation alleviates motor impairment in Parkinson’s disease: association with peripheral inflammatory regulatory T-cells and SYT6
Source: Mol Neurodegener. 2024 Oct 25;19:80. doi: 10.1186/s13024-024-00770-4 (PMC11515224; doi:10.1186/s13024-024-00770-4)
Supplement: Supplementary file 6 — Supplementary Material 6. [file 13024_2024_770_MOESM6_ESM.doc]

**Supplementary Table 2A**. Differentially expressed protein summary

| Compare group | Regulated type | fold change  > 1.2 | fold change  > 1.3 | fold change  > 1.5 | fold change  > 2 |
| --- | --- | --- | --- | --- | --- |
| PD/NS | Up-regulated | 264 | 138 | 66 | 19 |
|  | Down-regulated | 194 | 112 | 55 | 14 |
| dTreg/NS | Up-regulated | 706 | 417 | 178 | 45 |
|  | Down-regulated | 640 | 390 | 187 | 62 |
| dTreg/PD | Up-regulated | 315 | 139 | 51 | 18 |
|  | Down-regulated | 424 | 237 | 98 | 13 |
| dTreg/rTMS | Up-regulated | 145 | 42 | 21 | 10 |
|  | Down-regulated | 192 | 84 | 19 | 4 |
| rTMS /NS | Up-regulated | 592 | 327 | 139 | 41 |
|  | Down-regulated | 493 | 298 | 132 | 45 |
| rTMS /PD | Up-regulated | 281 | 135 | 63 | 20 |
|  | Down-regulated | 340 | 172 | 70 | 13 |

Filtered with threshold value of expression fold change and P value < 0.05

dTreg=PD+Treg block+rTMS

| **Supplemetary table 2B. Information of docking proteinsTLR4 and SYT6.** | | | |
| --- | --- | --- | --- |
| **Proetin** | **PDB ID** | **Chain** | **Pymol atoms** |
| TLR4 | 2Z64 | A | 2189 |
| SYT6 | / | B | 2184 |

| **Supplementary table 2C. Docking site information between chain A and chain B** | | |
| --- | --- | --- |
| **Position in chain A** | **Position in chain B** | **Dot distance of PyMol(unit)** |
| SER-102 | LYS-488 | 2.1 |
| PHE-75 | PHE-322 | 2.6 |
| SER-76 | ARG-321 | 2.1 |
| LEU-52 | ARG-321 | 2.9 |
| PRO-49 | ARG-321 | 3.3 |
| LYS-47 | PHE-319 | 2.3 |
| LYS-47 | ASP-266 | 3 |
| LYS-47 | ASP-260 | 3.3 |
| LYS-47 | ASP-318 | 3.4 |
| TYR-72 | TRP-493 | 2.6 |
| SEP-71 | ARG-386 | 2.7 |
| SEP-71 | ARG-386 | 3.4 |
| ASP-95 | ARG-386 | 3.3 |
| ASP-95 | ARG-386 | 3.4 |

(SER-102= Serine at position 102 of the chain A; PHE-75= Phenylalanine at position 75 of chain A; SER-76=Serine at position 76 of the chain A; LEU-52= Leucine at position 52 of chain A; PRO-49= Proline at position 49 of chain A; LYS-47= Lysine at position 47 of chain A; TYR-72= Tyrosine at position 72 of chain A; SER-71= Serine at position 71 of the chain A; ASP-95= Aspartic acid at position 95 of chain A; LYS-488= Lysine at position 488 of chain B; PHE-322= Phenylalanine at position 322 of chain B; ARG-321= Arginine at position 321 of chain B; PHE-319= Phenylalanine at position 319 of chain B; ASP-266= Aspartic acid at position 266 of chain B; ASP-260= Aspartic acid at position 260 of chain B; ASP-318= Aspartic acid at position 318 of chain B; TRP-493= Tryptophan at position 493 of chain B; ARG-386= Arginine at position 386 of chain B)
